# How facial masks alter the interaction of gaze direction, head orientation, and emotion recognition

**DOI:** 10.3389/fnins.2022.937939

**Published:** 2022-09-21

**Authors:** Lea Thomas, Christoph von Castell, Heiko Hecht

**Affiliations:** Department of Psychology, Johannes Gutenberg-Universität Mainz, Mainz, Germany

**Keywords:** emotion perception, facial expression recognition, gaze direction, head orientation, face masks

## Abstract

The COVID-19 pandemic has altered the way we interact with each other: mandatory mask-wearing obscures facial information that is crucial for emotion recognition. Whereas the influence of wearing a mask on emotion recognition has been repeatedly investigated, little is known about the impact on interaction effects among emotional signals and other social signals. Therefore, the current study sought to explore how gaze direction, head orientation, and emotional expression interact with respect to emotion perception, and how these interactions are altered by wearing a face mask. In two online experiments, we presented face stimuli from the Radboud Faces Database displaying different facial expressions (anger, fear, happiness, neutral, and sadness), gaze directions (−13°, 0°, and 13°), and head orientations (−45°, 0°, and 45°) – either without (Experiment 1) or with mask (Experiment 2). Participants categorized the displayed emotional expressions. Not surprisingly, masks impaired emotion recognition. Surprisingly, without the mask, emotion recognition was unaffected by averted head orientations and only slightly affected by gaze direction. The mask strongly interfered with this ability. The mask increased the influence of head orientation and gaze direction, in particular for the emotions that were poorly recognized with mask. The results suggest that in case of uncertainty due to ambiguity or absence of signals, we seem to unconsciously factor in extraneous information.

## Introduction

The coronavirus disease 2019 (COVID-19) pandemic has posed a global challenge of enormous magnitude causing high monetary and non-monetary costs and severely impacting physical as well as mental health worldwide (Pedrosa et al., 2020; Rajkumar, 2020; Vindegaard and Benros, 2020; Xiong et al., 2020; Brodeur et al., 2021; Wessels et al., 2022; World Health Organization [WHO], 2022). Mandatory contact restrictions and mask wearing inevitably affect the nature of our social interactions. Not only has the total number of daily face-to-face interactions decreased, but we often allow for larger interpersonal distance contrary to our natural preference (Welsch et al., 2020, 2021). Moreover, a mask frequently covers the lower part of the face, including the nose, mouth, and chin, and thereby deprives us of facial cues that are crucial for emotion recognition. Emotions are an inherent part of social interactions, and their causes, functions and consequences are interpersonally shaped (Parkinson, 1996; Van Kleef, 2009; Van Kleef et al., 2016). Thus, the quality and success of social interactions crucially depends on emotional competences including emotion recognition abilities (Lopes et al., 2004, 2005; Mayer et al., 2008). How well do we recognize emotions under such conditions of reduced cue availability? To answer this question, we distinguish between partial occlusion of the face and effects of face aversion. How does the complete absence of facial signals of the lower face due to mask wearing alter emotion perception? And how do more traditional restrictions, such as the altered visibility or salience of facial signals due to gaze or head deflection (a disruption of horizontal symmetry) affect emotion recognition? We have encountered the latter case all along. We are rarely confronted with faces perfectly aligned with our viewing direction. Only in portrait photos do faces gaze straight, but they are overwhelmingly chosen as stimuli in studies of emotion recognition – as opposed to faces viewed from the side and/or with averted gaze. The systematic occlusion of all facial features below the eyes, in contrast, is novel. In the current paper, we aim to settle these questions by first investigating emotion recognition under conditions of natural information reduction by varying gaze direction and head orientation, secondly by examining the additional influence of mask wearing, and finally by looking at potential interactions of such effects. We hypothesized that wearing a face mask alters the effects of gaze direction and head orientation.

### Emotion-specific emotion recognition

It can be assumed that such cue reductions affect emotion recognition to varying degrees depending on the displayed emotion, as each emotion has been associated with characteristic facial features, and respective facial areas that carry the information (Ekman, 2017). The present study includes the following four basic emotions, since these are found in almost all approaches: *anger*, *fear*, *sadness*, and *happiness* (Ortony and Turner, 1990; Tracy and Randles, 2011), all of which differ systematically from the baseline *neutral* facial expression. Each of these emotions has been related to a prototypical expression composed of signals from both the eye and the mouth region (Ekman and Friesen, 1978; Ekman, 2017). In addition to the availability of characteristic features, their distinctiveness and visual salience are also important for relative recognition advantages. The more distinctive and salient a facial feature is for a given emotion, the easier the latter can be recognized in isolation (Calvo and Nummenmaa, 2008). The recognition advantage for happiness, for example, is attributed to the distinctiveness and visual salience of the smiling mouth (Calvo and Nummenmaa, 2008; Calvo et al., 2014). For other emotions, an association with one facial region is less stringent. Indications of which facial areas are most diagnostic for the emotions investigated in this study are based on different approaches. Such approaches include tracking eye-movements during emotion recognition (e.g., Eisenbarth and Alpers, 2011; Schurgin et al., 2014), manipulating the visibility of facial information through different techniques (e.g., Smith et al., 2005; Nusseck et al., 2008; Blais et al., 2012; Wegrzyn et al., 2017), or presenting different facial parts in isolation (e.g., Calvo et al., 2014). Since these approaches differ in stimulus material and task conditions, differences are to be expected. The process of emotion recognition is probably different when the access of information is limited as compared to when all information is available and eye-movements are tracked. One facial manipulation technique is the Bubbles technique developed by Gosselin and Schyns (2001), in which faces are seen through a mask containing small holes of variable size, the so-called bubbles. Changing the location of the holes allows to identify the face region most relevant for the recognition of a given emotion. Using this technique, Blais et al. (2012), for instance, demonstrated that the mouth region is the most informative facial area for the discrimination of facial expressions. Wegrzyn et al. (2017) used a similar technique – sequentially uncovering a mask consisting of multiple tiles – but reported different results: Eyes and mouth were both important, and their relative importance depended on the emotion presented. Recognition of sad, fearful, and angry faces benefited from information about the upper face, recognition of happy and disgusted faces from information about the lower face. In sum, the literature on emotion recognition suggests a clear prioritization of the mouth region for happiness (e.g., Nusseck et al., 2008), and a clear prioritization of the eye region for anger (e.g., Bombari et al., 2013). As for sadness and fear, some evidence suggests that the eye region is more important than the mouth region for both sadness (e.g., Eisenbarth and Alpers, 2011) and fear (e.g., Bassili, 1979). Other authors suggest that eye and mouth regions are equally important for fear (e.g., Schurgin et al., 2014) as well as for sadness (e.g., Calvo et al., 2014).

How exactly these mimic signals are processed in emotion recognition – that is, the relative contribution of configural and featural information – is still up for debate. A study by Bombari et al. (2013) suggests that, in general, configural processing is more relevant than featural processing for emotion recognition, but their relative contribution differs among emotions.

Further clues as to how much the individual emotions are affected by covering the lower part of the face can be drawn from research on the impact of naturally occurring coverings such as a niq$\bar{a}$b (Fischer et al., 2012; Kret and de Gelder, 2012; Kret et al., 2021). In terms of emotion recognition performance, results showed that the recognition of sadness and happiness was clearly impaired when only seeing the eyes vs. the whole face. In contrast anger and fear were recognized equally well in both conditions (Kret and de Gelder, 2012). Note that in the eyes-only condition, the eyebrows were not visible, which is the case, however, when wearing a face mask. Moreover, emotion perception from the eyes was also influenced by the type of face covering when comparing a niqa$\bar{\text{a}}$b with a cap and a scarf or censoring black bars (Kret and de Gelder, 2012; Kret et al., 2021). This suggests that the impact of a face mask on emotion recognition may differ from that of a niqa$\bar{\text{a}}$b or other face coverings, since it is tied to a different affective context. We presume that, in general, also other contextual factors such as gaze direction gain more influence in emotion recognition the more the face is obscured.

### The impact of gaze direction and head orientation on emotion recognition

There is more to emotion perception than just the prototypical facial expression. Emotion is a complex, multimodal phenomenon which is influenced by contextual cues. Such cues can come from other perceptual modalities, such as voice (de Gelder and Vroomen, 2000), leading to multisensory interactions (Adams et al., 2010b,^2017^; Adams and Nelson, 2011). They can also come from other channels, remaining within the visual modality (Adams et al., 2010b,^2017^; Adams and Nelson, 2011), such as body posture (Aviezer et al., 2008; Hassin et al., 2013) or other *extraneous* cues. In particular, due to anatomical conditions of the head and the face, the perception of facial expressions is inseparably linked with the perception of gaze direction and head orientation, resulting in observable interaction effects. We will call this type of emotion perception, which is open to influences and capable of interactions, *integrative* emotion perception.

Within *integrative* emotion perception, gaze direction and head orientation play a key role. Gaze may act as an indicator of attention or as a behavioral component in the processing of facial expressions. Whereas direct gaze seems to hold attention on the face that is viewed, averted gaze seems to shift it away (Senju and Hasegawa, 2005; Bindemann et al., 2008). Attention facilitates face processing and averted attention impairs it (Senju and Hasegawa, 2005; Bindemann et al., 2008; McCrackin and Itier, 2019). There are studies reporting a more accurate recognition performance with direct gaze compared to averted gaze, which, however, also depends on other features of the performer, the observer, and the task (Bindemann et al., 2008; Campbell et al., 2017). Strategically, gaze can complement emotional expression based on an underlying shared meaning. As suggested by the *compound social cues approach*, a composite social cue stimulus is generated, which gains a processing advantage over more reduced signals (Adams et al., 2010a,^2017^; Adams and Nelson, 2011; Adams and Kveraga, 2015). According to the *shared signal hypothesis*, gaze direction and facial expression share underlying motivational tendencies of approach and avoidance (Adams et al., 2003, 2006; Adams and Kleck, 2005), and congruent pairings of gaze direction and facial expression are perceived as more intense and are processed more efficiently than incongruent pairings (Adams and Kleck, 2003, 2005; Adams and Franklin, 2009; Benton, 2010; Adams and Nelson, 2011). However, these interactions have been shown to be quite stimulus- and task-dependent (Bindemann et al., 2008; Ricciardelli et al., 2016; Caruana et al., 2019). Furthermore, gaze can also provide relevant contextual cues about target and source of an emotion, which is particularly significant in the context of threat signals, such as anger and fear (Adams and Franklin, 2009). Combinations of anger and direct gaze or fear and averted gaze are of greater ecological relevance than other possible combinations because they provide information about the target and the source of a threat. Such combinations may lead to both increased salience and more efficient processing (Adams and Kleck, 2003; Adams et al., 2003; Putman et al., 2006; Tipples, 2006; Adams and Franklin, 2009; Adams and Kveraga, 2015; El Zein et al., 2015). This could lead to recognition advantages, depending on the prevalence and nature of interaction effects. However, we are less interested to compare different combinations of facial expression and gaze direction, but rather focus on the effect of averted versus direct gaze on the ability to recognize emotions. Here gaze direction can act as an indicator of attention, rather than a behavioral component. The extent of integrative processing of gaze direction and facial expression appears to be modulated by signal discriminability, with greater interaction potential when facial expressions are less distinct (Ganel et al., 2005; Graham and LaBar, 2007, 2012).

With regard to the role of head orientation, it points to the likely focus of attention and thereby carries information about the personal relevance of signals (Hess et al., 2007; Bublatzky et al., 2017). Thus, the head can modulate the signal value and influence signal processing. Faces directed at the observer are perceived as more relevant compared to averted faces (Hess et al., 2007; Bublatzky et al., 2017), and the direction of the head can be assumed to be related to the signal strength of facial expressions as a function of how much to the side the head is turned and limits the visibility of the mimic signals. As far as the emotion recognition performance is concerned, however, the data on the influence of head orientation is less clear than that on the influence of gaze direction, at least as far as the half profile (±45°) is concerned. Hess et al. (2007) who compared decoding accuracy of facial expressions presented in frontal view and 3/4 profile view reported better recognition performance for anger and neutral expressions with a frontally oriented face compared to a laterally oriented face. They also found a tendency toward better recognition performance for fear with a laterally oriented face compared to a frontally oriented face. The recognition of happiness and sadness, in contrast, was not affected by head orientation (Hess et al., 2007). Comparing emotion recognition of facial expressions presented in frontal and in profile view, a previous study by Surcinelli et al. (2021) found that fear, anger, and sadness were better recognized in frontal view compared to profile view whereas there was no difference in the recognition of surprise, disgust, happiness, and neutrality. Taken together, head aversion generally tends to impair emotion recognition, depending on the emotion and depending on how far the head is turned to the side.

Gaze direction and head orientation are also perceptually interlinked, as perception of gaze direction involves the integration of head orientation and the position of the eyes relative to the head (Langton, 2000; Seyama and Nagayama, 2005; Loomis et al., 2008; West, 2013; Sweeny and Whitney, 2017). The processing of the relative eye position appears to be largely based on relational processing of different components of the eye region (e.g., iris-eccentricity, Todorović, 2006, 2009) rather than relying on configural processing of the entire face (Jenkins and Langton, 2003; Schwaninger et al., 2005; Harari et al., 2016). Head orientation is mainly estimated on the basis of the deviation of the head shape from bilateral symmetry, and the deviation of the nose orientation from the vertical center (Wilson et al., 2000). Note that turning the head also occludes areas of the face, which results in critical information loss with larger head rotations. When the orientation of the head is difficult to discern, nose orientation seems to be especially relevant (Wilson et al., 2000). Overall, human perception can provide relatively precise estimates of gaze direction and also head orientation, as long as the head is not deflected too much (Langton et al., 2000; Wilson et al., 2000; Symons et al., 2004). However, both lateral gaze and head deviations from the center are sometimes greatly overestimated (Anstis et al., 1969; Loomis et al., 2008; Otsuka et al., 2016; Alais et al., 2018). The accuracy of estimates is influenced by interaction effects between gaze direction and head orientation. Hecht et al. (2021) have found that when head orientation differs by more than 10° relative to gaze direction, gaze direction exerts a clear attraction effect on the perceived head orientation, that is, the perceived head orientation is shifted in the direction of the given gaze. In contrast, when gaze remains directed toward a frontal target, turning the head to the left or right pushes perceived gaze direction in the opposite direction, what is called a repulsion effect (Gamer and Hecht, 2007; Todorović, 2009; Hecht et al., 2021).

To date, emotional facial expression, gaze direction, and head orientation have rarely been investigated together with respect to interaction effects within emotion perception. Most studies exploring emotion recognition have only considered two of these variables while the third was kept constant. However, Ganel (2011) studied the relationship between the perception of facial expression and gaze direction while at the same time varying head orientation. What he found is that under such ecologically valid conditions – when all information from head and face are present as they are in everyday social interactions – neither did gaze direction interfere with the processing of facial expression, nor did the latter alter the processing of gaze direction (Ganel, 2011).

### The impact of facial masks on emotion recognition

Since the COVID-19 crisis, the mask has emerged as another influential factor with a versatile impact on facial perception and, in particular, emotion recognition. The mask impairs facial perception in quantitative and qualitative ways. Face masks impede face recognition and identification (Carragher and Hancock, 2020; Freud et al., 2020; Noyes et al., 2021) and cause a switch from a holistic to a more local, feature-based processing mode, in adults and children (Freud et al., 2020; Stajduhar et al., 2021). The impact of mask wearing on the recognition of basic emotions has by now been well researched. Most studies reported a general deterioration of emotion recognition accuracy by around 20% (Carbon, 2020; Grundmann et al., 2021; Marini et al., 2021; Noyes et al., 2021; Pazhoohi et al., 2021; Kim et al., 2022; McCrackin et al., 2022). In contrast, Calbi et al. (2021) observed only a rather negligible impairment of emotion recognition when they presented static facial expressions of anger, happiness, and neutral faces with or without a sanitary mask or a scarf. Mask wearing also had a negative impact on the confidence in one’s own assessment of presented emotional facial expressions (Carbon, 2020; Pazhoohi et al., 2021). Moreover, masks reduced the perceived intensity of displayed emotions and amplified emotions that had not been displayed (Pazhoohi et al., 2021; Tsantani et al., 2022).

The extent of recognition impairment by facial masks varies among the individual emotions and seems to be context-specific. Several studies found no or only a slight impairment in emotion recognition for fear (Carbon, 2020; Kim et al., 2022; McCrackin et al., 2022), and the strongest impairment for disgust (Carbon, 2020; Noyes et al., 2021; McCrackin et al., 2022). A severe impairment was also observed for sadness (Marini et al., 2021; Kim et al., 2022), anger (Kim et al., 2022; McCrackin et al., 2022), surprise (Kim et al., 2022), fear (Noyes et al., 2021) and happiness (Carbon, 2020). However, sometimes the recognition of happiness was surprisingly well preserved (Marini et al., 2021; Kim et al., 2022; McCrackin et al., 2022). Mask wearing altered the confusion patterns among different emotions, such that several emotions were misinterpreted as neutral, and anger, disgust, and sadness were more frequently confused with each other (Carbon, 2020; Kim et al., 2022).

### Aims and hypotheses

So far, the effects of masks on emotion recognition have typically been studied with frontal portraits of forward-looking faces and without consideration of interaction effects among the emotional signals and extraneous cues. To the best of our knowledge, this is the first study to examine the impact of mask wearing on integrative recognition of basic emotions while varying three social cues – facial expression, gaze direction, and head orientation. Thus, the aim of the current study is to explore how gaze direction, head orientation, and emotional facial expression interact with respect to emotion perception, and how these interactions are altered by wearing a face mask.

To address this aim, our first experiment investigated interaction effects within emotion recognition, which occur with uncovered faces. For this purpose, we presented static face stimuli from the *Radboud Faces Database* (*RaFD*) displaying five facial expressions in combination with three different angles of gaze direction and head orientation each. We recorded emotion recognition performance, perceived gaze direction, and perceived head orientation. In a second experiment, we examined the impact of mask wearing on these interaction effects by adding realistic masks to the face stimuli with all other parameters remaining unchanged.

Without mask, we expected a deterioration in emotion recognition with gaze and head deflection compared to straight gaze and frontal head, with stronger effects of head orientation. This prediction was based on the assumption that a direct gaze facilitates emotion recognition due to attention binding, as compared to an averted gaze. A frontal – compared to an averted – head should facilitate emotion recognition due to higher signaled relevance and maximum visibility of mimic signals. We also hypothesized that emotion recognition is generally impaired by mask wearing and happiness is most affected. We presumed the greatest impairment for happiness due to the unique visual saliency and high diagnostic value of the smiling mouth. Finally, we expected that the influence of gaze direction and head orientation increases when wearing a mask. We reasoned that mask wearing decreases discriminability and thereby increases ambiguity of the displayed emotions, resulting in a higher susceptibility of emotion perception to the extraneous cues of gaze direction and head orientation.

## Experiment 1

### Materials and methods

#### Design

We designed the study as a repeated-measures experiment with four within-subjects factors: *face model* (four levels: two female and two male models), *facial expression* (five levels: anger, fear, happiness, neutral, and sadness), *gaze direction* (three levels: left, centered, and right), and *head orientation* (three levels: left, frontal, and right). All factors were fully crossed, resulting in a total of 180 stimuli. We implemented this design as an online experiment on the online platform *SoSci Survey*^1^. Each subject judged all 180 factorial combinations in different random orders. The main dependent variable was emotion recognition performance. Perceived gaze direction, head orientation, valence, and arousal were gathered as control variables to check the manipulation of the independent variables and to assess the quality of our study. This was particularly important as we had implemented an online experiment with limited controllability of the experimental setting. The assessment of perceived gaze direction and head orientation enabled to verify whether participants picked up the actual changes of gaze direction and head orientation. These measures further provided a baseline to later clarify (Exp. 2) whether mask-induced changes are mainly mediated by changes in emotion perception or by changes in the perception of gaze direction and head orientation. Valence and arousal were recorded to make sure that the five facial expressions evoked distinguishable emotional responses in our subjects. Furthermore, the recording of valence and arousal also allowed to ascertain whether there are major differences in the display of the facial expressions between the models.

#### Participants

Fifty-three subjects participated voluntarily in this online study. Ten subjects (19%) chose to abort the experiment before completion, and four subjects were eliminated because they had failed to follow the instructions. Given the length of the experiment, we consider the drop-out rate to be quite acceptable for an online experiment. The experiment took 70 min even when carrying out the task promptly and without breaks. The resulting sample comprised 39 adults (32 female and 7 male) aged from 19 to 60 years (*M* = 25.67 years, *SD* = 7.40 years), 87% of which were students. All reported normal or corrected-to-normal vision. They were recruited by means of university mailing lists and different social media platforms. Psychology students of the University of Mainz received partial course credit for participation. In accordance with the Declaration of Helsinki, all subjects gave written informed consent and were debriefed after the experiment. The study was conducted in line with the ethical standards of the local ethics board of the Psychological Institute of Mainz University. Since voluntary participation on a fully informed basis and anonymity were assured, and there was no risk for physical stress or disadvantages due to group assignment, the research fell under the blanket approval of the ethics board.

#### Material

**FIGURE 1:**
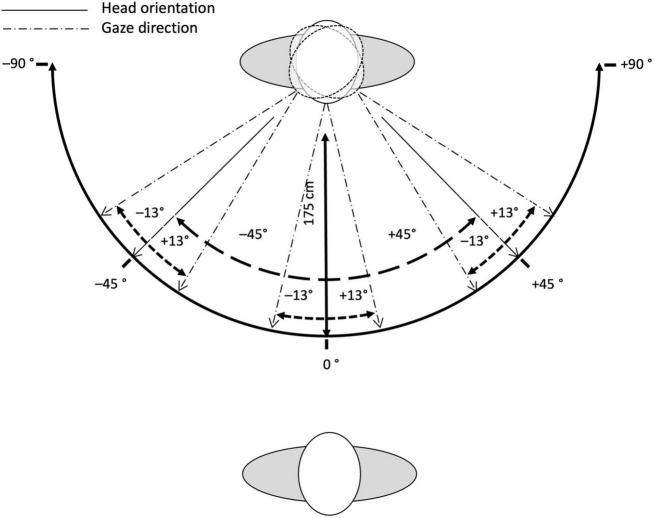
Schematic illustration of the variation of gaze direction and head orientation. Note that the models changed their gaze direction and facial expression. Head orientation was achieved with synchronized cameras at 0°, 45°, and –45° relative to frontal view.

**FIGURE 2:**
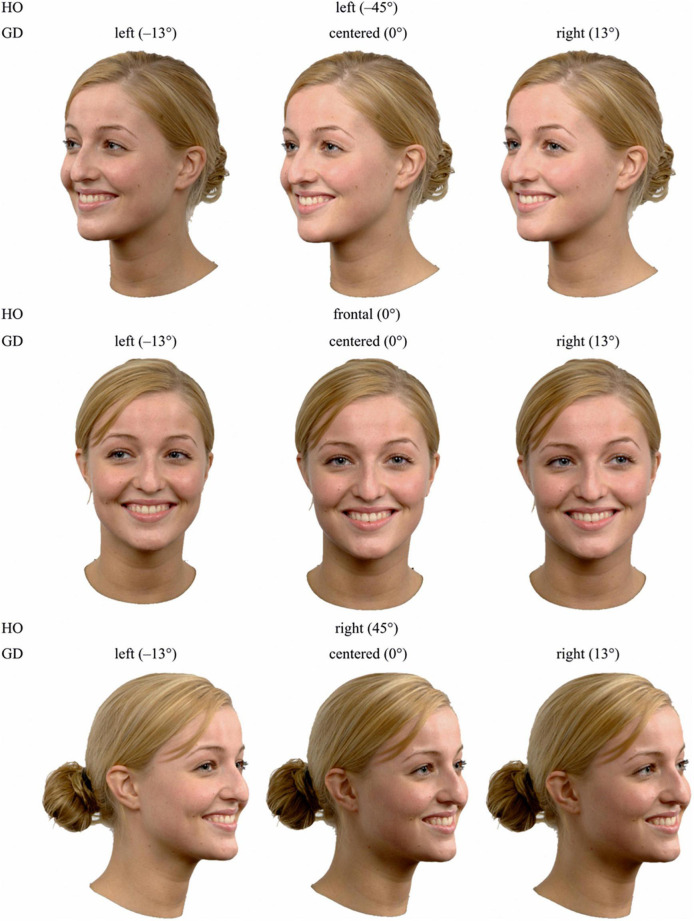
Example stimuli for uncovered faces, showing all nine possible combinations of gaze direction and head orientation for model 1 and the facial expression happiness. HO, head orientation; GD, gaze direction.

All face stimuli were obtained from the *Radboud Faces Database* (*RaFD*) (Langner et al., 2010). We used faces from four different Caucasian adults (two female: model 1, model 14, two male: model 20, model 23), each displaying five facial expressions (anger, fear, happiness, neutral, and sadness), paired with three different angles of gaze directions (left: −13°, centered: 0°, and right: 13°), and viewed from three different perspectives, which corresponded to three different angles of head orientation (left: −45°, frontal: 0°, and right: 45°). Figure 1 illustrates the interdependency between gaze direction and head orientation. Note that the pictures were taken simultaneously with a synchronized camera-array around the model, such that the exact same facial expression was photographed from all viewing angles (i.e., head orientations). This resulted in 180 face stimuli in total (4 models × 5 facial expressions × 3 gaze directions × 3 head orientations). The selection of the four models was based on the clarity and authenticity of the displayed facial expressions. All facial expressions from the *RaFD* were based on prototypes from the *Facial Action Coding System* (FACS; Ekman et al., 2002) and were monitored by FACS specialists during the photo shoot (Langner et al., 2010). Note that all four models had their mouth open and showed teeth when displaying happiness and, to a small extent, when displaying fear, but had their mouth closed when displaying neutral, anger and sadness. The original photographs were edited with *Photoscape*. Each image was scaled down to a resolution of 681 pixels × 570 pixels and cropped to remove background and upper body. Example stimuli are illustrated in Figure 2.

#### Procedure

Data collection took place between October 04 and November 30, 2020. Participants completed the online experiment by accessing a link, which they had received in advance. They were instructed to use a computer or a laptop, and all of them did so, with the exception of one subject who reported to have used a tablet. Before the experimental section started, they gave informed consent. During the experimental section, they were asked to assess facial stimuli as quickly and intuitively as possible in terms of (a) perceived gaze direction, (b) perceived head orientation, (c) displayed facial expression, and (d) valence and arousal. The time to respond was not limited. All ratings were made by click (mouse or touch). Each face stimulus was presented in color against a white background, centered in the middle of a single page, with the assessment tasks arranged around it. The two scales assessing gaze direction (left) and head orientation (right) were placed at the top, an emotion categorization task was placed at the bottom left, and the two scales assessing valence and arousal were placed at the bottom right (for an example page see Supplementary Material).

**FIGURE 3:**
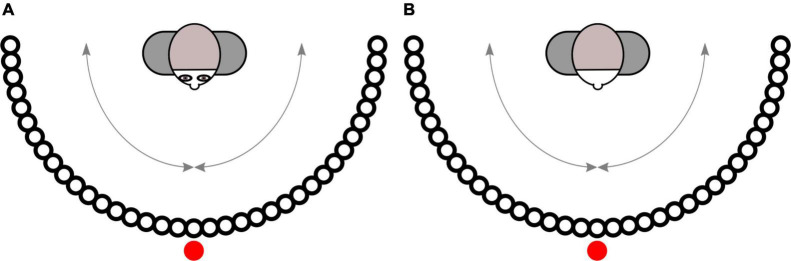
Svg-graphics for the assessment of gaze direction **(A)** and head orientation **(B)**.

Prior to the 180 experimental trials, all subjects completed the same training trial with one face stimulus, which was not part of the experimental stimulus set. The categorization of the displayed emotion was the main task and focus of interest. The remaining dependent variables primarily served as a manipulation check and to assess the quality of our online study. (a) and (b): Participants indicated perceived gaze direction and head orientation by means of two svg-graphics, which were internally created with the help of *Inkscape* (see Figure 3). The graphics each showed a person from a bird’s eye view surrounded by evenly spaced black dots arranged on a semicircle, each dot comprising 5° and the center of each dot marking a 5° step from 0° to 180°. A red dot indicated the position of the observer. Subjects had to select the circle that most closely matched the direction in which the displayed face was looking or pointing his or her head, respectively. (c): The displayed facial expressions were categorized by means of a single-choice task with eight response options. Participants indicated the emotional expression they believed to recognize in the face by selecting the most appropriate emotional label among the following eight options: *happiness*, *anger*, *sadness*, *contempt*, *disgust*, *neutral*, *fear*, and *surprise*. Three of these options represented distractors (*contempt*, *disgust*, and *surprise*). The response options were always presented in the same order to avoid errors that might have arisen when randomly switching their order, which would have introduced an additional processing demand. (d): Valence and arousal were recorded using visual analog scales ranging from *negative* to *positive* (valence), and from *calm* to *aroused* (arousal), respectively. Face stimuli and scales all appeared at the same time and remained on screen until all ratings had been completed and the subject navigated to the next page. Following the experimental trials, participants were asked to provide demographic data, and to answer several questions regarding personality traits as well as behaviors and experiences in the context of non-verbal communication (for the exact questions and answers see Supplementary Material). Finally, participants were debriefed and had the opportunity to receive partial credit for their participation. In total, the experiment lasted about 70 min.

### Results

We first report the results for emotion recognition, then we give a short overview of the control variables.

To investigate the influence of the displayed facial expression, gaze direction, and head orientation on emotion recognition performance, we conducted a 5 × 3 × 3 repeated-measures analysis of variance (rmANOVA; univariate approach) with emotion (anger, fear, happiness, neutral, and sadness), gaze direction (left, centered, and right), and head orientation (left, frontal, and right) as within-subject factors. The rate of correctly recognized emotional facial expressions served as the dependent variable. These values were aggregated across the four models. Subsequently, we calculated four further rmANOVAs with the same factorial design for the additional dependent measures judged gaze direction, head orientation, valence, and arousal.

All tests were performed at a significance level of α = 0.05. We performed power analyses using G*Power (Faul et al., 2007). In all subsequent rmANOVAs, a sample size of 39 was sufficient to achieve a power of over 80% at an alpha of 5% for each reported effect. Where indicated, we used the Greenhouse-Geisser correction for the degrees of freedom (correction factor ε; this correction was applied to all subsequent ANOVAs). As a *post hoc* analysis, we conducted univariate rmANOVAs with the same factorial design separately for each emotion or pairwise comparisons, which were corrected according to Hochberg (1988) to account for multiple testing. Prior to conducting pairwise comparisons, the differences between the paired values were routinely analyzed for normality of distribution by using Shapiro–Wilk tests (see Supplementary Material). In some cases, the normality assumption was violated. For reasons of consistency, however, Wilcoxon signed-rank tests were then calculated for all pairwise comparisons. For all corresponding data sets, additionally to means and SDs, medians (Mdns) and 95% confidence intervals (CIs) are reported.

#### Emotion recognition

##### Basic emotion recognition

**FIGURE 4:**
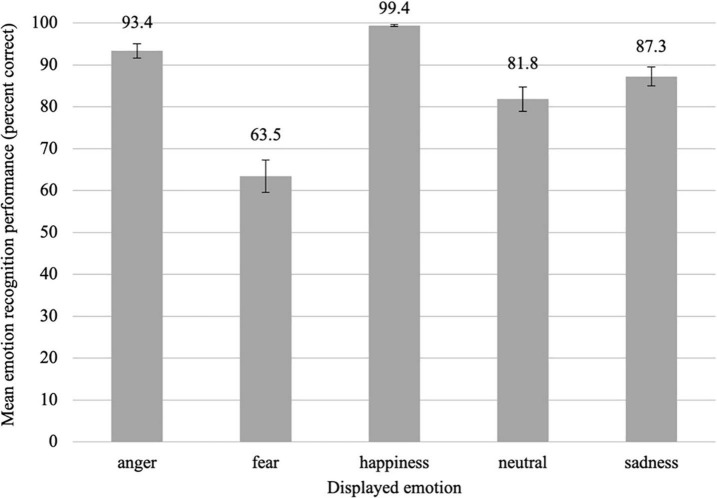
Mean emotion recognition performance in percent correct for uncovered faces as a function of the displayed emotion, averaged across all gaze directions and head orientations. Error bars indicate the standard error of the mean (SEM) of the 39 individual data points in each condition.

Overall, participants recognized emotional facial expressions from unmasked faces with a recognition rate of 85.1% (*SD* = 8.3%), which was clearly above the chance level of 12.5%. Recognition performance, however, differed depending on the emotion presented, with a clear recognition advantage for happiness and a clear recognition disadvantage for fear (see Figure 4; anger: *M* = 93.4%, *SD* = 10.8%, fear: *M* = 63.5%, *SD* = 24.1%, happiness: *M* = 99.4%, *SD* = 1.3%, neutral: *M* = 81.8%, *SD* = 18.4%, sadness: *M* = 87.3%, *SD* = 14.0%). Overall, we found the following rank order of emotion recognition performance: *happiness* > *anger* > *sadness* > *neutral* > *fear* (see Figure 4).

In line with these observations, the main effect of emotion was significant, *F*(4,152) = 33.38, *p* < 0.001, $\eta_{p}^{2}$ = 0.468, ε = 0.68. According to the *post hoc* tests, all emotions except for neutral and sadness, *z* = −1.21, *p_*corr*_* = 0.227, *r* = 0.19, differed significantly from each other in the direction of the rank order shown in Figure 4, all |*z*| ≥ 2.70, *p_*corr*_ ≤* 0.014, *r* ≥ 0.43 (for more details see Supplementary Material).

Emotion recognition performance also varied depending on the idiosyncrasies of the models. For instance, fear was particularly poorly recognized in the female model 1 and in the male model 20.

##### Integrative emotion recognition

**FIGURE 5:**
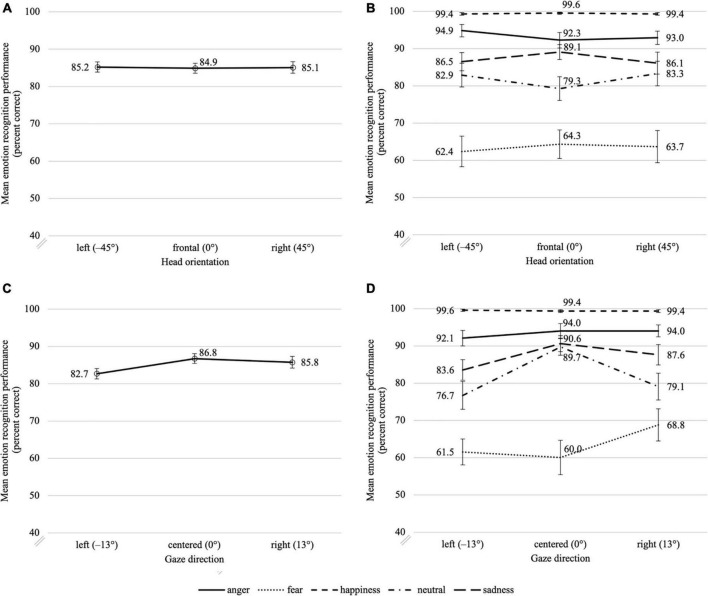
Mean emotion recognition performance in percent correct for uncovered faces as a function of head orientation (top row, **A,B**) and gaze direction (bottom row, **C,D**), aggregated across all facial expressions (left column, **A,C**) and additionally as a function of the five different facial expressions (right column, **B,D**). Error bars indicate the standard error of the mean (SEM) of the 39 individual data points in each condition.

The effects of gaze direction and head orientation were less obvious. Overall, emotion recognition performance with averted gaze or head was almost as good as with centered gaze and frontally aligned head, with the exception of a small drop in recognition performance for leftward gaze (see Figures 5A,C). The rmANOVA showed a significant main effect of gaze direction, *F*(2,76) = 10.75, *p* < 0.001, $\eta_{p}^{2}$ = 0.220. According to the *post hoc* tests, emotion recognition was significantly reduced with left gaze than with centered gaze, *z* = −3.45, *p_*corr*_* = 0.002, *r* = 0.55, as well as with right gaze, *z* = −3.32, *p_*corr*_* = 0.002, *r* = 0.53; right gaze and centered gaze did not differ significantly from each other, *z* = −1.06, *p_*corr*_* = 0.290, *r* = 0.17. The main effect of head orientation was clearly not significant, *F*(2,76) = 0.07, *p* = 0.935, $\eta_{p}^{2}$ = 0.002.

As can be seen in Figures 5B,D, the effects of gaze direction and head orientation varied in size and direction depending on the displayed emotion. Neutral, sadness, and fear appeared to be most affected. Note that, averaged across all gaze directions, anger and neutral tended to be recognized even better with averted head than with frontal head, and, averaged across all head orientations, fear tended to be recognized even better with averted gaze than with centered gaze.

In the rmANOVA, the emotion × gaze direction interaction was significant, *F*(8,304) = 7.81, *p* < 0.001, $\eta_{p}^{2}$ = 0.170, ε = 0.58, while the emotion × head orientation interaction was not significant, *F*(8,304) = 1.67, *p* = 0.139, $\eta_{p}^{2}$ = 0.042, ε = 0.66. To examine the emotion × gaze direction interaction in more detail, we calculated separate rmANOVAs for each emotion. These *post hoc* tests showed that gaze direction significantly affected the recognition performance for the three most poorly recognized emotions: fear, *F*(2,76) = 5.88, *p* = 0.004, $\eta_{p}^{2}$ = 0.134, neutral, *F*(2,76) = 16.29, *p* < 0.001, $\eta_{p}^{2}$ = 0.300, and sadness, *F*(2,76) = 5.61, *p* = 0.005, $\eta_{p}^{2}$ = 0.129, with neutral being the most affected. Happiness, *F*(2,76) = 0.14, *p* = 0.870, $\eta_{p}^{2}$ = 0.004, and anger, *F*(2,76) = 1.32, *p* = 0.274, $\eta_{p}^{2}$ = 0.033, were not significantly affected. The strong effect for the neutral faces can possibly be attributed to the fact that neutral as non-emotional facial expression is rarely ‘perfect’ in the sense of a complete absence of mimic signals, but rather to a greater or lesser extent, depending on the model, contains emotion-specific mimic signals. This might increase ambiguity, which is tried to be solved by seeking further information from other social dimensions. In sum, these results suggest that emotion recognition remained at comparable levels across the manipulation of gaze direction and head orientation with some variation due to changes in gaze direction in performance for all emotions but happiness and anger. Thus, the effect of gaze direction on emotion recognition appears to be modulated by the degree of discriminability of the displayed emotion, with more ambiguous emotions being more likely to be influenced by gaze direction. *Post hoc* tests showed that fear was detected better with averted gaze than with centered gaze, whereas neutral and sadness were recognized better with centered gaze than with averted gaze, whereby the effect of gaze direction was symmetrical only for neutral (see Table 1).

**TABLE 1:** Experiment 1: Results of the Wilcoxon signed-rank tests for correct emotion recognition (percentages given as decimals) (*N* = 39).

Comparison	* ${}_{\bar{\text{Δ}}}$ *	*Mdn* (*x_1_ − x_2_*)	95% CI	*z*	*p*_*corr*_	*r*
**Fear**						
Gaze direction						
Left – centered	0.01	0.00	[−0.04, 0.07]	−0.45	0.654	0.07
Right – centered	0.09	0.00	[0.03, 0.14]	−2.97	0.009	0.48
Left – right	−0.07	−0.08	[−0.13, −0.02]	−2.46	0.028	0.39
**Neutral**						
Gaze direction						
Left – centered	−0.13	−0.08	[−0.19, −0.07]	−3.86	<0.001	0.62
Right – centered	−0.11	−0.08	[−0.16, −0.06]	−3.61	<0.001	0.58
Left – right	−0.02	0.00	[−0.06, 0.02]	−1.06	0.288	0.17
**Sadness**						
Gaze direction						
Left – centered	−0.07	0.00	[−0.12, −0.02]	−2.84	0.015	0.45
Right – centered	−0.03	0.00	[−0.07, 0.01]	−1.65	0.099	0.26
Left – right	−0.04	0.00	[−0.08, 0.00]	−2.14	0.064	0.34

Pearson’s *r* is reported as a measure of effect size.

#### Control variables

Since we did not observe any surprising significant phenomena that were relevant for our research question, the results of the analysis of perceived gaze direction and head orientation are reported only briefly. Both gaze direction and head orientation were correctly interpreted by the participants according to the instructions and estimated quite well overall. For perceived gaze direction, we found a slight leftward bias, for perceived head orientation, we observed a slight rightward bias. Since both biases appeared across models, we assume that the position of the assessment scales (left: gaze direction and right: head orientation) had caused these distortions.

In line with our expectations and previous research (Hecht et al., 2021), but surprisingly limited to the frontal head orientation, we found a repulsion effect of head orientation on perceived gaze direction as well as an attraction effect of gaze direction on perceived head orientation (the data can be found in the Supplementary Material). Perceived gaze was misestimated to diverge more from the centered head orientation than was actually the case (repulsion effect). At the same time a given gaze direction pulled the judged head orientation toward the gaze (attraction effect). However, for averted head orientation, the effects of gaze direction and head orientation changed or did not occur at all: For perceived gaze direction the repulsion effect changed into an attraction effect, but for the perceived head orientation the attraction effect was no longer detectable under such conditions. Those interaction patterns were basically the same for all five facial expressions.

Concerning perceived valence and arousal, the facial expressions were perceived as differently pleasant and arousing according to a constant rank order from high to low across all gaze directions and head orientations (valence: *happiness* > *neutral* > *fear* > *sadness* > *anger*; arousal: *fear* > *anger* > *happiness* > *sadness* > *neutral*).

Overall, the data of the control variables indicate that our online experiment, despite its inherent limitations, yielded estimates of average head orientation and average gaze direction at the same accuracy levels (within a few degrees) as those obtained previously in related laboratory experiments (Hecht et al., 2021).

## Experiment 2

### Materials and methods

#### Design

This experiment was a perfect replication of Exp. 1 with the only difference that the models wore a facial mask. The latter was photoshopped into the original photographs. We used the same repeated measures design as in the first experiment, using the same factors and dependent variables as before. Thus, in the combined analysis, the between-factor of mask emerged (Exp. 1: without mask, Exp. 2: with mask) leading to a mixed design with facial expression, gaze direction, and head orientation as within-subject factors, and with *mask* as between-subject factor.

#### Participants

A total of 71 subjects participated in this online study. 14 subjects (20%) dropped out early and seven subjects were eliminated because they failed to follow the instructions. Another five subjects were excluded because they had already participated in the first experiment. Again, we consider the drop-out rate to be quite acceptable for an online experiment lasting more than 1 h. This resulted in a final sample size of 45 adults (31 female and 14 male) aged from 18 to 53 years (*M* = 26.67 years, *SD* = 7.95 years), 93% of which were students. All indicated normal or corrected-to-normal vision. They were briefed and debriefed as before. Recruitment procedure and compensation were identical to Exp. 1.

#### Material

**FIGURE 6:**
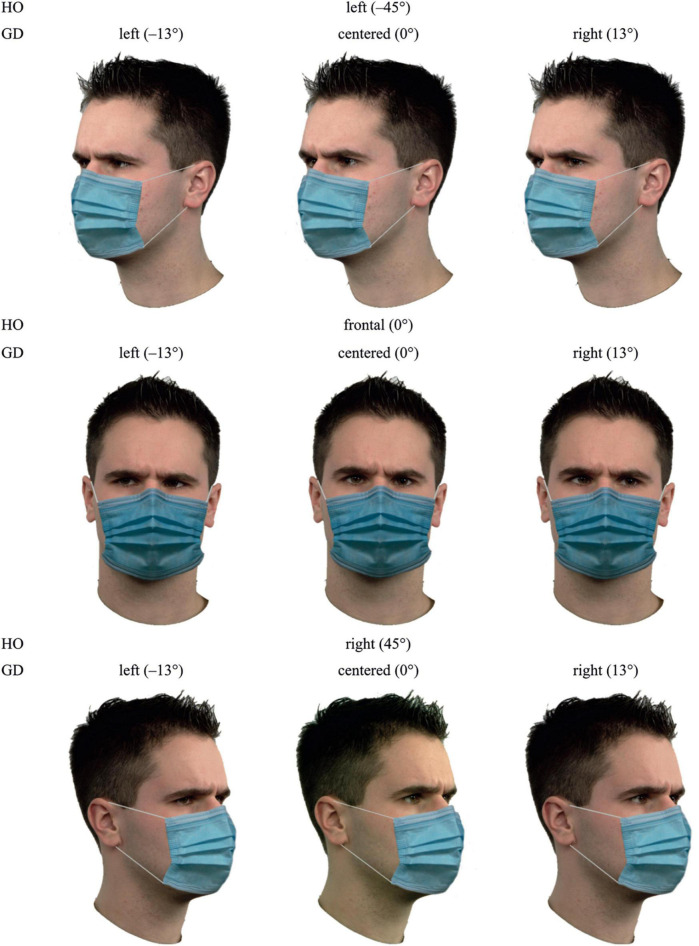
Example stimuli for masked faces, showing all nine possible combinations of gaze direction and head orientation for model 20 and the facial expression anger. HO, head orientation; GD, gaze direction.

The face stimuli were the same stimuli as in the first experiment, except that a face mask was superimposed on each face using *GIMP* (*GNU Image Manipulation Program*). For this purpose, we photographed a surgical face mask from angles corresponding to the displayed head orientations of the face stimuli (−45°, 0°, and 45°). Then, the photographs were cropped to remove background and original ear loops (see Supplementary Material), graphically adjusted to the individual faces, and complemented with matching ear loops. The image height of the stimuli remained unchanged while the image width was extended to 400 pixels. Example stimuli are illustrated in Figure 6.

#### Procedure

The online experiment was identical to the first experiment, except that the mask had been added to the face stimuli, and participants were additionally asked whether they had already participated in the first experiment. Accordingly, the experiment also lasted about 70 min. All participants used a computer or laptop as instructed, except for one, who used a smartphone. Data collection took place between March 18 and May 31, 2021.

### Results

We first report the results for emotion recognition, then we give a short overview of the results for the control variables.

In order to investigate the influence of mask wearing on participants’ emotion recognition performance as well as on the interactions between displayed facial expression, gaze direction, and head orientation within emotion recognition, we calculated a 2 (mask) × 5 (emotion) × 3 (gaze direction) × 3 (head orientation) mixed ANCOVA using the same within-subjects factors as before, and adding the between-subjects factor mask (Exp. 1: without mask and Exp. 2: with mask) as well as the covariate of participant gender to account for slightly different gender distributions in Exp. 1 (∼82% females) and Exp. 2 (∼69% females). To further investigate mask interactions, we conducted a rmANOVA for masked stimuli in an analogous manner to the rmANOVA in Exp. 1 to be able to directly compare the effects for uncovered and masked stimuli. For each control variable reported below, we conducted a mixed ANCOVA using the same factorial design as for emotion recognition performance.

#### Emotion recognition

##### Basic emotion recognition

**FIGURE 7:**
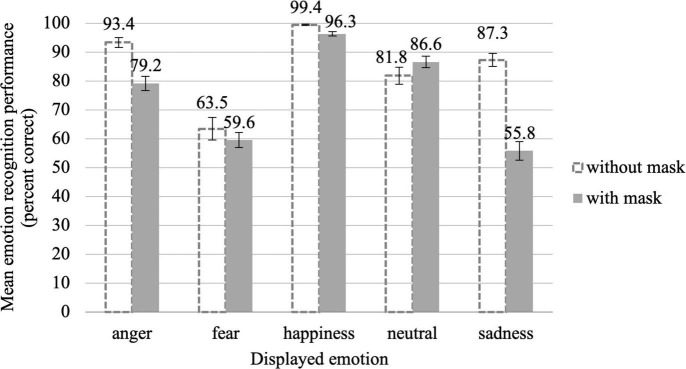
Mean emotion recognition performance in percent correct for uncovered faces (dashed bars; Exp. 1) and masked faces (filled bars; Exp. 2) as a function of the displayed emotion, averaged across all gaze directions and head orientations. Error bars indicate the standard error of the mean (SEM) of the 39 and 45 individual data points per displayed emotion, respectively.

With mask, the overall emotion recognition rate dropped to 75.5% (*SD* = 8.7%), amounting to a deterioration of almost 10%. Figure 7 illustrates the emotion recognition performance for masked faces compared to uncovered faces as a function of the displayed emotion. It is important to note that the recognition rate for all emotions remained clearly above the chance level of 12.5%. Taking a closer look at Figure 7, it becomes evident that the mask deteriorated recognition performance for all facial expressions except for neutral, for which it even slightly improved it. Obviously, the reduction of visible mimic signals is not necessarily always adverse for emotion recognition but can also sometimes protect against overinterpretation. It appears very plausible that in the case of a neutral facial expression, which is characterized by the absence of mimic signals, a reduction of visible facial regions can have a beneficial effect. What is surprising, however, is the very strong deterioration for sadness, with losses of about 30%, as well as a strong deterioration for anger, with losses of about 15%. This changed the rank order of emotion recognition performance from *happiness* > *anger* > *sadness* > *neutral* > *fear* (Exp. 1) to *happiness* > *neutral* > *anger* > *fear* > *sadness* (Exp. 2). In line with these observations, the mixed ANCOVA revealed a significant main effect of mask, *F*(1,81) = 25.35, *p* < 0.001, $\eta_{p}^{2}$ = 0.238, and a significant mask × emotion interaction, *F*(4,324) = 18.08, *p* < 0.001, $\eta_{p}^{2}$ = 0.182, ε = 0.82.

The rmANOVA for masked stimuli showed that the effect of the displayed emotion on recognition performance, *F*(4,176) = 62.85, *p* < 0.001, $\eta_{p}^{2}$ 0.588, ε = 0.74, was somewhat more pronounced than for uncovered faces ($\eta_{p}^{2}\,$ = 0.468). *Post hoc* tests showed that, with mask, all presented emotions except for fear and sadness, *z* = −0.77, *p*_*corr*_ = 0.444, *r* = 0.11, differed significantly from each other in recognition performance, all |*z*|≥ 2.59, *p_*corr*_ ≤* 0.020, *r* ≥ 0.39 (for more details see Supplementary Material).

Thus, wearing a mask led to a reduction in overall emotion recognition performance, notwithstanding some variation in performance decrement depending on the displayed emotion and the idiosyncrasies of the models, with emotion and model each exerting a greater influence compared to unmasked faces. For instance, we found particularly poor recognition of sadness in the female model 14.

##### Integrative emotion recognition

**FIGURE 8:**
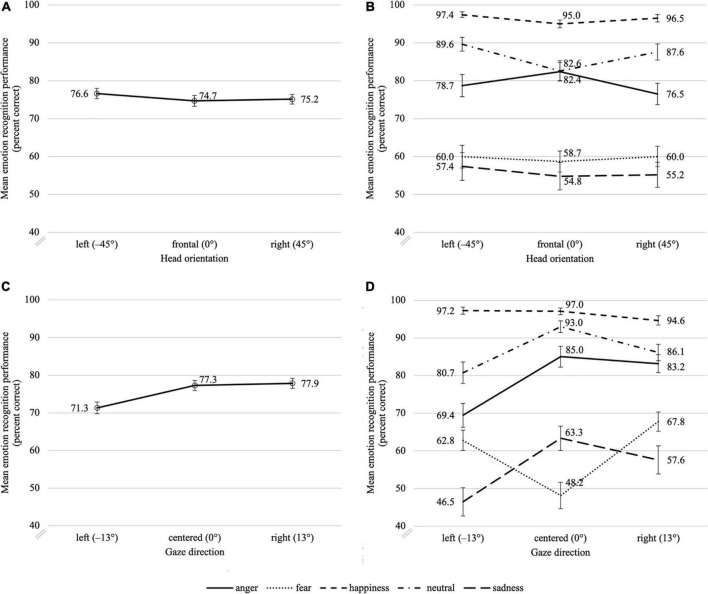
Mean emotion recognition performance in percent correct for masked faces as a function of head orientation (top row, **A,B**) and gaze direction (bottom row, **C,D**), aggregated across all facial expressions (left column, **A,C**) and additionally as a function of the five different facial expressions (right column, **B,D**). Error bars indicate the standard error of the mean (SEM) of the 39 and 45 individual data points in each condition, respectively.

The effect of head orientation on emotion recognition remained weak, but the mask had reversed the direction of this effect for all emotions except neutral, and now all emotions except anger tended to be better recognized with averted head as compared to frontal head (see Figures 8A,B).

In the mixed ANCOVA, the mask × head orientation interaction, *F*(2,162) = 1.34, *p* = 0.264, $\eta_{p}^{2}$ = 0.016, and the mask × emotion × head orientation three-way interaction were not significant, *F*(8,648) = 1.45, *p* = 0.192, $\eta_{p}^{2}\,$ = 0.018, ε = 0.76, which indicates that mask wearing did not significantly change the effect of head orientation on emotion recognition performance. Note, however, that in the rmANOVA for masked stimuli both the main effect of head orientation, *F*(2,88) = 3.14, *p* = 0.048, $\eta_{p}^{2}\,$ = 0.067, and the emotion × head orientation interaction, *F*(8,352) = 2.72, *p* = 0.016, $\eta_{p}^{2}\,$ = 0.058, ε = 0.71, were significant, though small. For the main effect of head orientation, the *post hoc* tests showed no significant differences between the individual head orientations, all |*z*|≤ 2.06, *p_*corr*_ ≥* 0.117, *r* ≤ 0.31 (for more details see Supplementary Material). The separate *post hoc* rmANOVAs for the individual emotions revealed that, with mask, head orientation had a significant effect on recognition performance for anger, *F*(2,88) = 4.18, *p* = 0.019, $\eta_{p}^{2}$ = 0.087, happiness, *F*(2,88) = 3.22, *p* = 0.045, $\eta_{p}^{2}\,$ = 0.068, and neutral, *F*(2,88) = 7.87, *p* = 0.002, $\eta_{p}^{2}\,$ = 0.152, ε = 0.79. Fear, *F*(2,88) = 0.26, *p* = 0.774, $\eta_{p}^{2}$ = 0.006, and sadness, *F*(2,88) = 0.68, *p* = 0.508, $\eta_{p}^{2}$ = 0.015, were not significantly affected. Going further into detail, *post hoc* tests showed that happiness and neutral were better recognized with averted head orientation, while there were no significant differences between the head orientations for anger (see Table 2). The effect of gaze direction had clearly gained influence, especially for the emotions that were most poorly recognized with mask: sadness, fear, and anger (see Figures 8C,D). Furthermore, with mask, the rank-order changed depending on gaze direction, whereas, without mask, it had remained constant across all head orientations and gaze directions. In line with this, in the mixed ANCOVA the mask × gaze direction interaction just missed significance, *F*(2,162) = 3.02, *p* = 0.052, $\eta_{p}^{2}\,$ = 0.036, in combination with a clearly significant mask × emotion × gaze direction three-way interaction, *F*(8,648) = 6.82, *p* < 0.001, $\eta_{p}^{2}\,$ = 0.078, ε = 0.72. This indicates that the effect of gaze direction on emotion recognition performance changed as a function of both mask wearing and displayed emotion. In the rmANOVA for masked stimuli, the main effect of gaze direction, *F*(2,88) = 29.67, *p* < 0.001, $\eta_{p}^{2}\,$ = 0.403, was more prominent than for the uncovered faces ($\eta_{p}^{2}$ = 0.220). Comparable with the results of Exp. 1, emotion recognition was significantly reduced with left gaze than with centered gaze, *z* = −4.80, *p_*corr*_* < 0.001, *r* = 0.72, as well as with right gaze, *z* = −5.45, *p_*corr*_* < 0. 001, *r* = 0.81, and right gaze and centered gaze did not differ significantly from each other, *z* = −0.25, *p*_*corr*_ = 0.803, *r* = 0.04. The rmANOVA also showed a significant emotion × gaze direction interaction, *F*(8,352) = 24.12, *p* < 0.001, $\eta_{p}^{2}$ = 0.354, ε = 0.66, which was also more pronounced than for the uncovered stimuli ($\eta_{p}^{2}$ = 0.170). The *post hoc* rmANOVAs run separately for the individual emotions showed that, in addition to the effects already found in Exp. 1 for fear, *F*(2,88) = 31.82, *p* < 0.001, $\eta_{p}^{2}$ = 0.420, ε = 0.85, sadness, *F*(2,88) = 22.32, *p* < 0.001, $\eta_{p}^{2}$ = 0.337, and neutral, *F*(2,88) = 21.54, *p* < 0.001, $\eta_{p}^{2}$ = 0.329, ε = 0.81, gaze direction now also had a significant effect on the recognition performance for anger, *F*(2,88) = 29.74, *p* < 0.001, $\eta_{p}^{2}$ = 0.403, and, thus, now for all emotions except happiness, *F*(2,88) = 2.76, *p* = 0.069, $\eta_{p}^{2}$ = 0.059. Note that this pattern is compatible with the assumption that the influence of gaze direction increases with rising uncertainty. Thus, the deteriorating effect of mask wearing on the recognition performance for anger could explain why, with mask, the effect of gaze direction had also reached significance for this emotion. According to the *post hoc* tests, only fear was recognized better with averted gaze, while anger, neutral, and sadness were better recognized with centered gaze, whereby the effect of gaze direction was now symmetrical for all emotions except anger (see Table 3).

**TABLE 2:** Experiment 2: Results of the Wilcoxon signed-rank tests for correct emotion recognition (percentages given as decimals) (*N* = 45).

Comparison	* ${}_{\bar{\text{Δ}}}$ *	*Mdn* (*x_1_ − x_2_*)	95% CI	*z*	*p*_*corr*_	*r*
**Anger**						
Head orientation						
Left – frontal	−0.04	0.00	[−0.07, 0.00]	−1.78	0.150	0.27
Right – frontal	−0.06	−0.08	[−0.11, −0.01]	−2.36	0.054	0.35
Left – right	0.02	0.08	[−0.02, 0.06]	−1.14	0.256	0.17
**Happiness**						
Head orientation						
Left – frontal	0.02	0.00	[0.01, 0.04]	−2.60	0.027	0.39
Right – frontal	0.01	0.00	[−0.01, 0.04]	−1.31	0.308	0.20
Left – right	0.01	0.00	[−0.01, 0.03]	−1.02	0.308	0.15
**Neutral**						
Head orientation						
Left – frontal	0.07	0.00	[0.03, 0.11]	−3.52	<0.001	0.52
Right – frontal	0.05	0.00	[0.01, 0.09]	−2.10	0.072	0.31
Left – right	0.02	0.00	[−0.01, 0.05]	−1.41	0.160	0.21

Pearson’s *r* is reported as a measure of effect size.

**TABLE 3:** Experiment 2: Results of the Wilcoxon signed-rank tests for correct emotion recognition (percentages given as decimals) (*N* = 45).

Comparison	* ${}_{\bar{\text{Δ}}}$ *	*Mdn* (*x*_1_ − *x*_2_)	95% CI	*z*	*p*_*corr*_	*r*
**Anger**						
Gaze direction						
Left – centered	−0.16	−0.17	[−0.20, −0.11]	−4.96	<0.001	0.74
Right – centered	−0.02	0.00	[−0.06, 0.02]	−0.91	0.361	0.14
Left – right	−0.14	−0.08	[−0.18, −0.09]	−4.64	<0.001	0.69
**Fear**						
Gaze direction						
Left – centered	0.15	0.17	[0.09, 0.20]	−4.08	<0.001	0.61
Right – centered	0.20	0.17	[0.14, 0.25]	−4.97	<0.001	0.74
Left – right	−0.05	−0.08	[−0.09, −0.01]	−2.45	0.014	0.37
**Neutral**						
Gaze direction						
Left – centered	−0.12	−0.08	[−0.16, −0.08]	−4.55	<0.001	0.68
Right – centered	−0.07	−0.08	[−0.10, −0.04]	−4.15	<0.001	0.62
left – right	−0.05	0.00	[−0.10, −0.01]	−2.43	0.015	0.36
**Sadness**						
Gaze direction						
Left – centered	−0.17	−0.17	[−0.22, −0.12]	−4.70	<0.001	0.70
Right – centered	−0.06	−0.08	[−0.11, −0.01]	−2.01	0.045	0.30
Left – right	−0.11	−0.08	[−0.16, −0.06]	−3.61	<0.001	0.54

Pearson’s *r* is reported as a measure of effect size.

#### Control variables

There were no significant changes in perceived gaze direction or head orientation due to wearing a mask. With regard to emotion perception, this suggests that the observed changes in interaction effects for masked faces are mediated by changes in emotion perception and are not due to a shift in the perception of gaze direction and head orientation.

Overall, face stimuli with mask were perceived as more pleasant and more arousing than those without mask. Perceived valence increased for all emotions except happiness, for which it slightly decreased. The mask increased perceived arousal for all presented emotions except for anger, there it decreased arousal. The rank orders for both perceived valence and arousal remained basically unchanged by the mask.

## Discussion

Emotional facial expressions are basically defined and, in most cases, studied based on frontally aligned faces with straight gaze. However, in everyday social interactions both head orientation and gaze direction vary considerably. Turning the head sideways interrupts face symmetry and alters the visible information from facial cues such as eyebrows, eyes, nose, and mouth. Depending on their shape, size, and configuration, some features are more affected by self-occlusion than others. Averting the gaze, on the other side, also interrupts face symmetry and changes various characteristics of the eyes such as iris-eccentricity and the visible part of the sclera. Mask wearing further reduces visibility of facial features, which may remove important remaining cues in the averted face. The impact of facial masks had thus far been investigated mainly using frontally aligned faces with straight gaze. The aim of our study was to evaluate the impact of face masks on integrative emotion recognition with varying gaze direction and head orientation.

In our first experiment, we have examined interaction effects between facial expression and the extraneous cues gaze direction and head orientation that come into play during emotion recognition with uncovered faces. In the second experiment, we have then investigated how these interaction effects are altered by wearing a face mask. The findings from Exp. 1 thereby offer insight into effects of naturalistic variation of cue visibility and saliency due to head and gaze deflection on emotion recognition of uncovered faces. The results from Exp. 2 shed light on the additional effects of mask-induced occlusion of facial cues. Thus, the second experiment also addresses the interaction between two types of cue reduction: altered cue visibility of the whole face and complete absence of cues of the lower face.

With respect to emotion recognition under non-masked conditions (Exp. 1), we found an overall accuracy rate of around 85%, which is comparable to that of 82% found in the validation study of the *RaFD* (Langner et al., 2010). It should be noted, however, that for the latter only images with frontal head orientation were considered, all eight facial expressions of the *RaFD* were assessed (in addition: *surprise, disgust*, and *contempt*), and there were no distractors to choose from. Because of the averted faces in two thirds of our trials, the task was harder, but emotion recognition remained at a high level. In fact, the accuracy rate of emotion recognition was superior to that reported for uncovered faces in some (e.g., Grundmann et al., 2021) but not all other studies (Carbon, 2020; Marini et al., 2021). In line with previous research, we observed a clear recognition advantage for happiness and a recognition disadvantage for fear (e.g., Calvo and Lundqvist, 2008); the other emotions ranged somewhere in between. Compared to the findings from Langner et al. (2010) for frontal head orientation, the recognition rates we obtained in the first experiment were quite similar for happiness and neutral, even slightly better for anger and sadness (about 7–8%), but significantly worse for fear (about 20%). General differences were to be expected since we had presented only four models but three head orientations. Regarding the large difference in the recognition of fear, we strongly assume that due to the similarity of the facial expressions fear and surprise, the inclusion of surprise has led to its confusion with fear. Note that surprise featured as a distractor response option but was never displayed in the stimuli.

The effect – or rather the null-effect – of face aversion is striking: Head orientation relative to the observer had no significant effect on emotion recognition. Emotions were detected just as well with the head faced 45° sideways as with a frontally oriented head. Thus, on the one hand, the visibility of mimic signals is less compromised at 45° than expected, and on the other hand, we seem to be able to flexibly adapt our emotion recognition strategies, for example, by changing the relative weighting of individual signals depending on their visibility. This assumption is in line with findings of a study by Stephan and Caine (2007) on face recognition. They noted that, while identity-specific information from the nose and mouth is fairly robust across head orientations, information from the eyes is more susceptible to view transformations, but the availability of information from the eyes suffers largely only at head orientations beyond 45°.

Gaze direction *per se* had a significant effect on emotion recognition and – contrary to our expectations – influenced emotion recognition more strongly than did head orientation. However, this effect was smaller than expected, what might be attributable to the more ecologically valid conditions that included variable head orientations. This is consistent with findings by Ganel (2011), who observed a lower interaction potential between facial expression and gaze direction when simultaneously varying head orientation. Interestingly, in our study, the emotions that were most poorly recognized were most affected. These results are consistent with the assumption that when uncertainty arises due to a lack of signal clarity, other information such as gaze direction is used for clarification (Ganel et al., 2005; Graham and LaBar, 2007, 2012). Thus, unambiguous emotions appear to be processed without gaze interference, but ambiguous emotions tend to be modulated in their processing by gaze direction. With the exception of fear, the results lend support to our hypothesis of a general recognition advantage with direct gaze, as explicable via attentional binding (Senju and Hasegawa, 2005). This corresponds well with the findings of Campbell et al. (2017) and McCrackin and Itier (2019). The fact that fear without mask was better detected with averted gaze, which cannot be explained by general attention binding across emotions, shows that gaze direction at least for this emotion has to be taken into account when investigating emotion recognition. We suggest that for fear, a prioritized processing of threat signals overrides the effect of attentional binding. Whereas a direct gaze generally facilitates emotion recognition through attentional binding, in the case of fear, a lateral gaze would thus signal more danger and therefore increase perceptual sensitivity. Averted gaze might thereby improve the recognition as a function of sclera exposure, as suggested by Carlson and Aday (2018).

What do our findings imply for the impact of mask wearing? Our data from the second experiment indicate a significant overall reduction in emotion recognition of almost 10% due to partial occlusion by the face mask. The deterioration is smaller compared to most other studies that have examined emotion recognition impairment by masks (Carbon, 2020; Grundmann et al., 2021; Marini et al., 2021; Noyes et al., 2021; Pazhoohi et al., 2021; Kim et al., 2022; McCrackin et al., 2022). This discrepancy may be caused by differences in stimulus material or task conditions, which would be compatible with the relatively high emotion detection rate we observed for unmasked faces. However, there may be also other reasons: First, the timing of the data collection could be relevant; since the beginning of the COVID-19 pandemic compensation strategies could have been learned. Second, varying all three variables at the same time provided more ecologically valid conditions, which may have allowed for less restricted emotion recognition. We found this impairment of emotion recognition for all facial expressions except neutral, which was even better recognized with the mask. As for neutral expressions, our results compare favorably with those reported by Carbon (2020) and Marini et al. (2021), who neither observed improvement nor impairment in this case. It is plausible that for the recognition of a neutral facial expression a reduction of visible mimic signals can be beneficial, since we are prone to *emotion overgeneralization* (Zebrowitz et al., 2010). The conclusion that masking of less important facial areas can improve emotion recognition has also been drawn by Kim et al. (2022), who observed an improvement in emotion recognition for happiness when covering the eyes with sunglasses.

With regard to the other emotions, we did not observe the strongest deterioration with happiness (∼3%), as expected, but rather with sadness (∼30%) and anger (∼15%). This strong impairment of sadness and anger recognition is remarkable because it is contrary to theoretical assumptions and empirical findings. However, similar results have been reported in previous studies. A comparable strong mask impairment for sadness and anger was observed by Kim et al. (2022). Marini et al. (2021) also found the strongest impairment for sadness, albeit less pronounced compared to our results. Carbon (2020) has shown that, although sadness was not most affected by the mask, confidence in one’s own assessment for sadness decreased the most. Especially in times of the pandemic, overlooking and misinterpreting facial expressions of sadness can be grave. Given the relatively well expressed mimic signals of the face stimuli and the distinctive mimic signals theoretically present in the eye region for both anger and sadness (Ekman and Friesen, 1978), these observations suggest that the relevant signals from the eye region are not used efficiently and that the recognition of sadness and anger in everyday life might depend more on the lower face than usually assumed. A study by Blais et al. (2012) noted that people generally use the mouth more than the eyes for emotion differentiation in basic emotions and that the eye region was insufficiently taken into account compared to an ideal observer. Another explanation for this emotion-specific mask impairment could also be that it is linked to the processing mode of the individual emotions, since mask wearing appears to interrupt holistic processing (Freud et al., 2020; Stajduhar et al., 2021). Consequently, the emotions most impaired would be those that rely more heavily on holistic or configural processing for recognition. There is evidence that anger and sadness are actually processed more on the basis of configural information (Bombari et al., 2013). Happiness, in contrast, can also be well processed on the basis of featural information (Bombari et al., 2013). Anger, however, as opposed to sadness, may still have a detection advantage due to prioritized processing of threat signals. The recognition of happiness was merely slightly impaired, maybe due to the fact that happiness was the only positive emotion among all those presented. We assume that it is easier to distinguish emotions according to valence than to recognize the specific emotion. It is also possible that the typical wrinkles on the outer edge of the eyes, which distinguish a genuine *Duchenne* smile from a social smile (Ekman, 2017), may be more indicative of happiness than previously assumed. At least, the results suggest that, in the case of happiness, we can switch our detection strategy and adapt to the conditions of reduced visual signals. A similar robustness of happiness was also observed in other studies (Marini et al., 2021; Kim et al., 2022; McCrackin et al., 2022). In contrast, Carbon (2020) found a stronger impairment in emotion recognition of happiness, although happiness was the only positive emotion investigated.

What might also contribute to the emotion-specific pattern of recognition impairment observed in our study is that sadness, anger, and fear are most susceptible to be mixed up by untrained observers since they all involve eyebrow movements. Such movements are sometimes difficult to distinguish and can look quite different depending on the person, due to individual differences such as the shape of the eyebrows. In addition, mimic signals also convey a wide variety of non-emotional information (Adams and Nelson, 2011; Knapp et al., 2014), for example about thought processes, especially involving the upper face (Rinn, 1984). The absence of information about the lower face increases ambiguity. In sum, available signals are not necessarily used optimally, which may not be a challenge until we are confronted with unfamiliar conditions such as wearing masks. Conversely, this also means that there is a lot of potential for learning.

With regard to the interaction between the mask and the extraneous cues gaze direction and head orientation, we found remarkable results. For masked faces, the influence of both gaze direction and head orientation on emotion recognition increased. Again, the emotions that were most poorly recognized – with mask different ones than for unmasked faces – were most affected by gaze direction, as evidenced by a significant mask × emotion × gaze direction three-way interaction. Thus, in line with our hypothesis, in the case of decreased discriminability and increased ambiguity of the displayed emotions the extraneous cues gained more influence. However, note that the increase for head orientation was too small to produce any significant mask interaction in the mixed ANCOVA, although it caused a significant main effect of head orientation as well as a significant emotion × head orientation interaction in the rmANOVA for masked stimuli. It is also important to emphasize that our data show that factoring in such additional information not necessarily improves the performance but can also impair it even more. As for gaze direction, emotion recognition tended to deteriorate with gaze deflection for all emotions except fear. Thus, in case of uncertainty, we seem to unconsciously integrate extraneous information into our emotion processing regardless of whether or not it is helpful.

As for head orientation, the increased influence cannot be explained with the reduction of ambiguity alone. The change in signal strength in the eye region during head rotation might become more influential when the information of the mouth-nose region is lacking, and therefore may also contribute to the greater impact of head orientation. Stephan and Caine (2007) reported that, in terms of identity recognition, the change in the visibility of information from the eyes with head rotation only affects recognition performance when the head is turned more than 45° sideways, but the story might be different in terms of emotion recognition. Thus, it remains unclear to what extent the face occlusion exerts its influence via the altered signal visibility and to what extent via reassignment of signal relevance during cue integration as necessitated by mask wearing. A reassignment or reweighting would be supported by the fact that, with mask, all emotions except anger tended to be better recognized with averted head than with frontal head. In contrast, without mask, such tendencies were only observable for anger and neutral. We suppose that up to an angle of 45° head rotation, the visibility of the signals changes so slightly that it does not significantly affect emotion recognition, but clearly alters the relative salience of certain signals from the eye region. The typical wrinkles in the eye area associated with happiness are mainly located at the outer edge of the eyes and may be more exposed when viewed from the side. In the prototypical expression of anger, in contrast, the eyebrows are lowered and contracted, shifting the focus of the signals to the center between the eyes, which is probably less salient with the head turned sideways as compared to the head facing forward. Consequently, our results suggest that, overall, the mask has the greatest effect on emotion recognition when the head is facing frontally and that mask-induced impairment is attenuated under ecologically valid conditions when the head can turn freely.

To conclude, mask wearing not only impaired emotion recognition in an unexpected emotion-specific way, but it also altered interaction effects between facial expressions and extraneous cues both quantitatively and qualitatively.

### Limitations, implications, and recommendations

There are some limitations, of which the most important will be pointed out. We consider the type of manipulation of gaze direction and head orientation, which is tied to the stimulus material of the *RaFD*, as the major limitation of this study. This is because these two variables do not vary independently of each other. Gaze direction is attached to and defined in dependence of head orientation. It always shifts at the same deviation angle of about 13° relative to the head, while the head rotates at a 45° angle. Since we had also selected only one head deviation angle (−45°, 0°, and 45°), there is neither a continuous gradation of head orientation nor of gaze direction or of the relative distance between both. Thus, individual effects cannot be considered as a function of a continuous change in those parameters, which should be kept in mind regarding interpretation and generalizability. Our results suggest that more head and gaze angles should be tested in future research.

Another shortcoming of our study is the restriction to four face models. We found that in some models certain emotions were particularly poorly recognized, which suggests that the models differed in facial features relevant to emotion recognition, as is also to be expected with different faces in everyday life. However, given that the emotions were acted and given that for practical reasons, a larger or representative sample of actors was prohibitive, differences among the models cannot be interpreted. Many factors could be responsible for such differences, for instance invariant features such as eye color and shape or eye and pupil distance, or variable features like mimic movements. Even though the models had been trained by FACS experts (Facial Action Coding System) and the displayed facial expressions had been validated (Langner et al., 2010), they still differ in their emotional expressions as well as in their neutral expression (Jaeger, 2020). Since the eye region is considered the most variable facial area (Itier and Batty, 2009), model differences could be particularly relevant when faces are covered by a mask. This would be consistent with our finding that mask wearing led to more variation in emotion recognition performance between models.

Furthermore, this study is subject to the inherent limitations of any online experiment. It did not allow for tight experimental control of viewing distance and monitor resolution, and potential distractions during the experiment. This might have added noise to the data, however, we have no indication that this noise could have been systematic. Other limitations are associated with the use of photographs with posed facial expressions and superimposed face masks. Enacting emotional expressions may be the only way to produce a large database, however, in everyday life, we may be confronted with expressions that go beyond an actor’s ability, that are less intense or fragmentary (subtle emotions), or that merely last for a fraction of a second (microexpressions). Moreover, emotional facial expressions are usually not the focus of attention. In real life, the effects of mask wearing on emotion recognition may therefore be more pronounced. Or they may be compensated by adaptation strategies both in sending and receiving emotional facial signals – or by integrating more other emotional cues such as body posture. Especially sadness seems to be very well recognizable on the basis of body posture (de Gelder and Van den Stock, 2011), which in daily social interactions could compensate for the strong impairment observed in this study. Adaptation strategies as a consequence of mask wearing have been observed by Kulke et al. (2021), who reported a slight improvement in emotion recognition from the eyes, which was, however, limited to women. Also, Okazaki et al. (2021) found a stronger eye involvement in smiling (as measured by orbicularis oculi activity) when wearing a mask. In contrast, Levitan et al. (2022) observed that masked positive faces were rated as less positive than unmasked positive faces regardless of whether the whole face or only the upper face was presented, which they attributed to reduced positive emotion and/or reduced expressivity of positive emotion as a consequence of wearing a mask. Further studies investigating such adaptation processes will be necessary.

May the high proportion of females be a problem? There is a consistent female advantage in recognizing emotions in particular when the face is partially covered (Grundmann et al., 2021). Moreover, men tend to look more often and longer at the mouth and especially at the nose during emotion recognition (Vassallo et al., 2009), whereas women tend to look more at the eyes (Hall et al., 2010). This would even suggest a greater mask impairment in males and thus more pronounced gender differences with masked faces. It is therefore reasonable to assume that in gender-balanced samples, the effects we have found could only be more pronounced.

Insights into the emotion-specific recognition impairment by masks broadens our understanding of how efficiently available mimic signals can be used in intuitive emotion perception by untrained observers. This allows for purposive emotional training to strengthen those emotional competences that are particularly affected by wearing a mask. The results of our study indicate that facial expression, gaze direction, and head orientation are closely linked at the perceptual level and that their simultaneous inclusion can make a difference. Therefore, future studies might benefit from a design that simultaneously considers all three variables. Further studies will be necessary to gain insights into adaptation to and compensation of the cue reduction caused by mandatory mask wearing. They should explore whether and how long such adaptation strategies will persist after the pandemic crisis.

### Conclusion

The results of our study add to and qualify the existing body of literature on the impact of mask wearing on emotion recognition. It is indispensable to take into account gaze direction and head orientation as extraneous cues highly relevant in facial perception in real-life situations. Emotion recognition was surprisingly well adapted to the altered visibility of facial signals due to head and gaze deflection, with gaze direction only slightly influencing the emotions that were most poorly recognized. However, when the facial signals of the lower face were completely absent due to mask wearing, emotion recognition was clearly impaired, with sadness and anger being the most affected emotions. Moreover, the mask also amplified the influence of gaze direction and head orientation. Thus, when there is increased uncertainty due to ambiguity or absence of signals, extraneous cues are more likely to be integrated in the perceptual process of judging emotion from facial features.

## Data availability statement

The original data presented in this study are included in the article/Supplementary material, further inquiries can be directed to the corresponding author.

## Ethics statement

The study was conducted in line with the ethical standards of the local ethics board of the Psychological Institute of Mainz University. Since voluntary participation on a fully informed basis and anonymity were assured, and there was no risk for physical stress or disadvantages due to group assignment, the research fell under the blanket approval of the ethics board. The participants provided their written informed consent to participate in this study. Written informed consent was obtained from the individual(s) for the publication of any identifiable images or data included in this article.

## Author contributions

HH, CC, and LT designed the experiments and edited the manuscript. LT implemented and conducted the experiments, analyzed the data, and drafted the manuscript. All authors contributed to the article and approved the submitted version.

## Footnotes

1
https://www.soscisurvey.de
