# Supplementary material for: How facial masks alter the interaction of gaze direction, head orientation, and emotion recognition
Source: Front Neurosci. 2022 Sep 21;16:937939. doi: 10.3389/fnins.2022.937939 (PMC9533556; doi:10.3389/fnins.2022.937939)
Supplement: Supplementary file 8 [file Data_Sheet_8.docx]

Supplementary Material

# Supplementary Figures and Tables

## Supplementary Figures


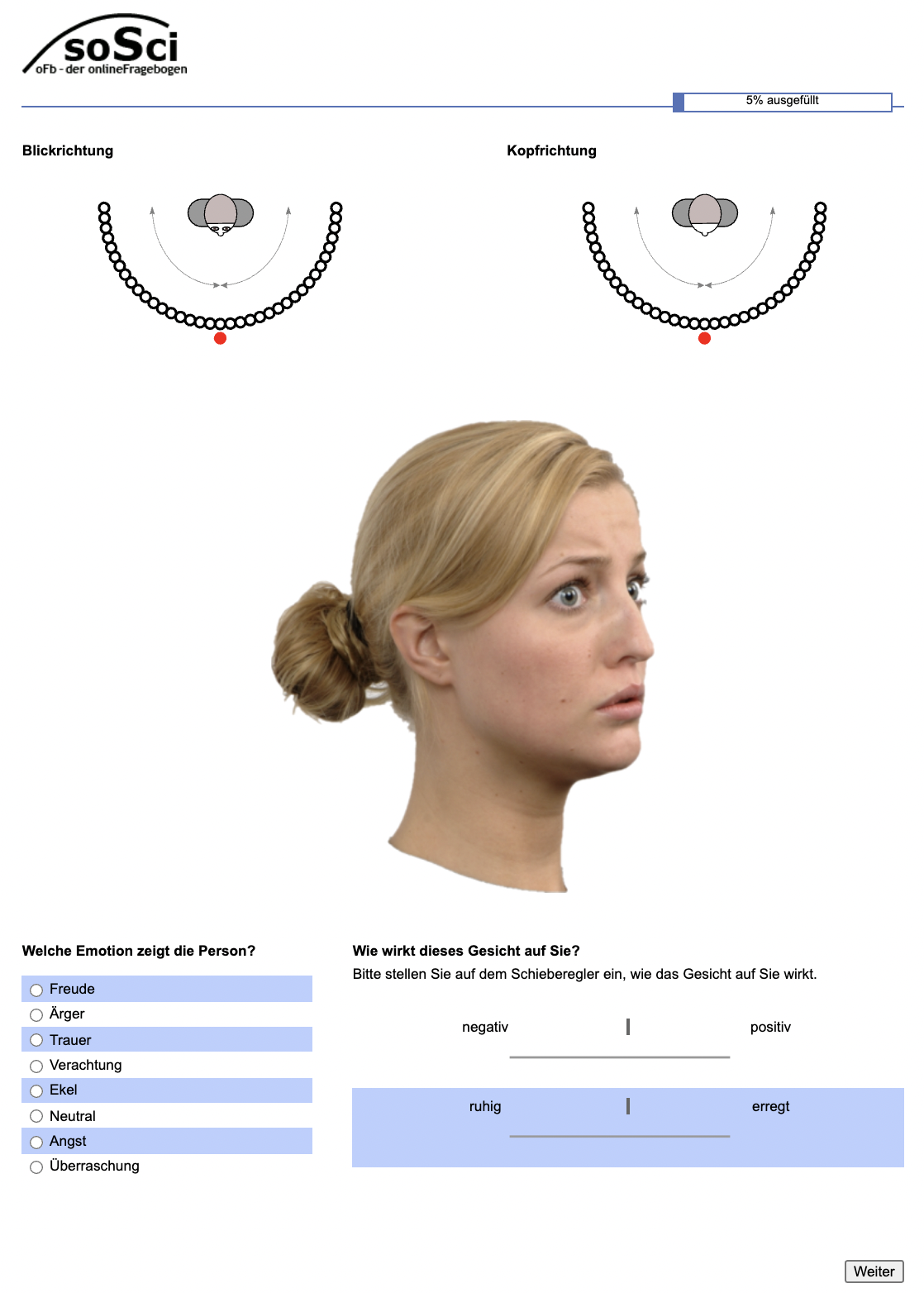


**Supplementary Figure S1.** Sample page of the online experiment with a face stimulus from the first experiment.

|  | gar nicht  [not at all] | wenig  [a bit] | mittelmäßig  [moderately] | überwiegend  [mostly] | völlig  [completely] |
| --- | --- | --- | --- | --- | --- |
| Für wie offen halten Sie sich?  [How open do you consider yourself to be?] |  |  |  |  |  |
| Für wie gewissenhaft halten Sie sich?  [How conscientious do you consider yourself to be?] |  |  |  |  |  |
| Für wie extrovertiert halten Sie sich?  [How extroverted do you consider yourself to be?] |  |  |  |  |  |
| Für wie umgänglich halten Sie sich?  [How agreeable do you consider yourself to be?] |  |  |  |  |  |
| Für wie ängstlich halten Sie sich?  [How anxious do you consider yourself to be?] |  |  |  |  |  |
| Für wie emotional halten Sie sich?  [How emotional do you consider yourself to be?] |  |  |  |  |  |
| Für wie aufmerksam halten Sie sich in Ihrer allgemeinen Wahrnehmung?  [How attentive do you consider yourself to be in your general perception?] |  |  |  |  |  |
| Wie wichtig ist Blickkontakt für Sie generell?  [How important is eye contact for you in general?] |  |  |  |  |  |
| Wie wichtig ist Ihnen, dass Ihr Gegenüber Sie während einer Unterhaltung anschaut?  [How important is it to you that the other person looks at you during a conversation?] |  |  |  |  |  |
| Wie wichtig ist Ihnen, dass ihr Gegenüber sich Ihnen zuwendet, auch wenn er/sie Sie nicht direkt anschaut?  [How important is it to you that the person you are talking to turns to you, even if he/she is not looking directly at you?] |  |  |  |  |  |

**Supplementary Figure S2.** Questions asked at the end of the online experiment regarding personality traits as well as behaviors and experiences in the context of nonverbal communication. Note: English translations of the German questions (in brackets) were not presented during the experiment and have been added post-hoc.

[mostly]

[completely]

[moderately]

[a bit]

[not at all]


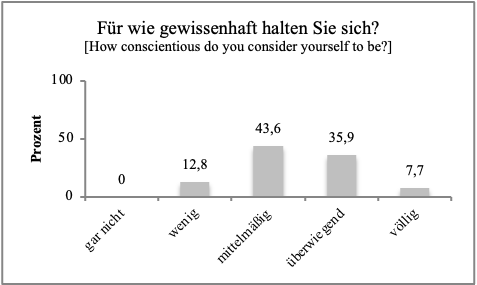

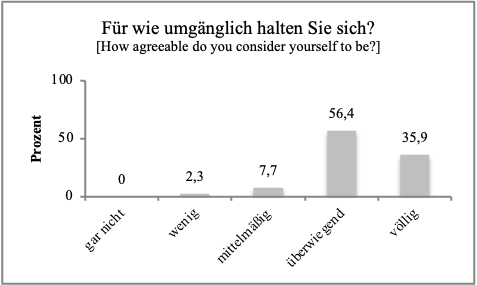

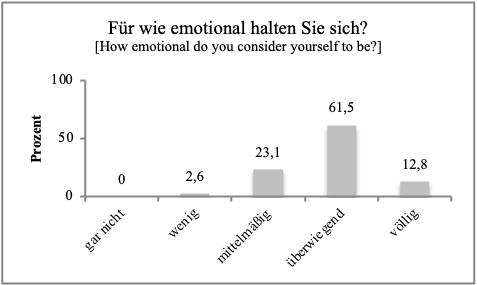

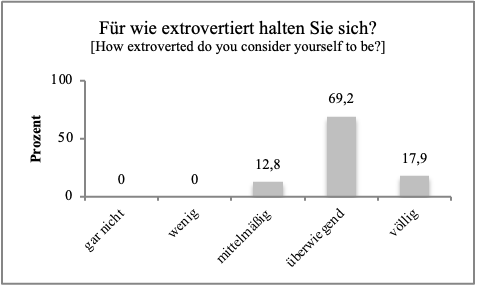

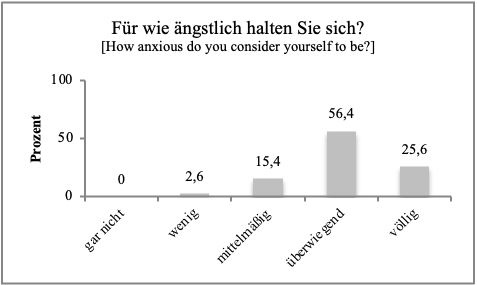

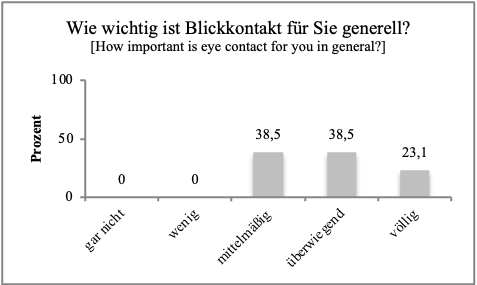

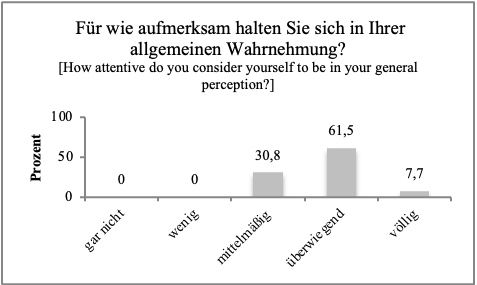

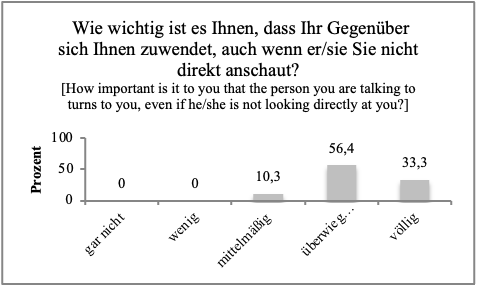

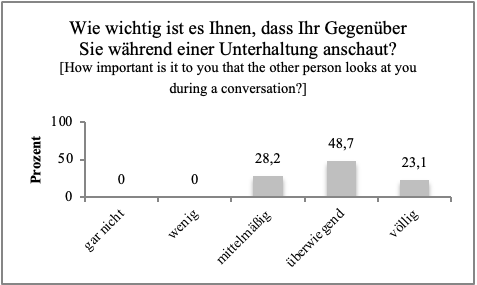

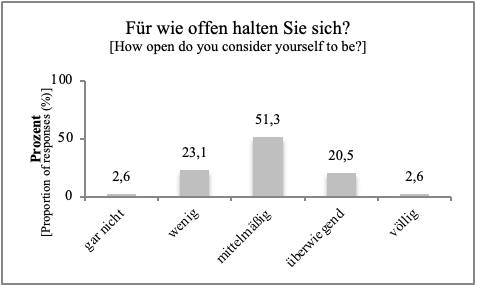


**Supplementary Figure S3.** Experiment 1: Answers to the personal questions.

###

**Supplementary Figure S4.** The three basic mask orientations.

[Proportion of responses (%)]

[mostly]

[a bit]

[not at all]

[completely]

[moderately]


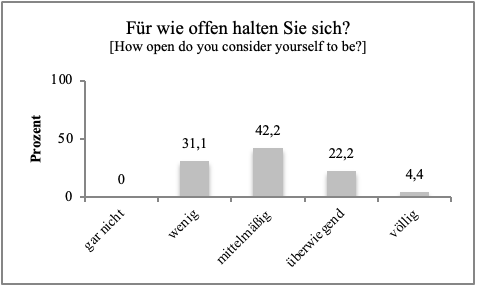

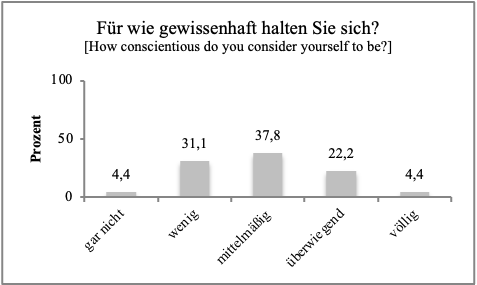

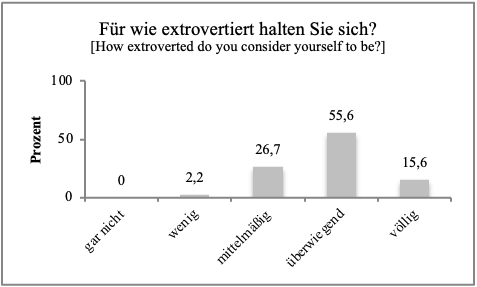

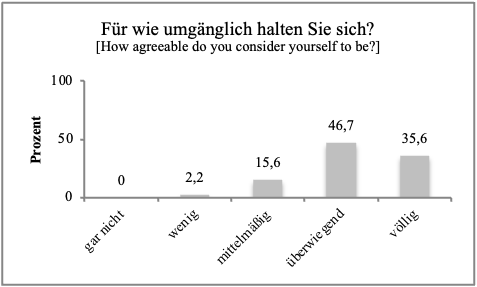

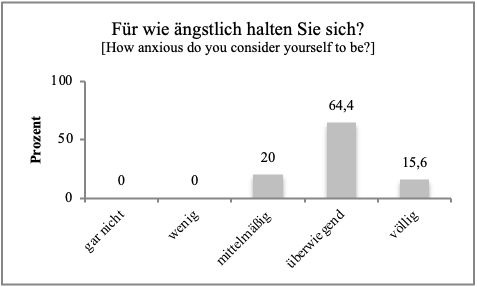

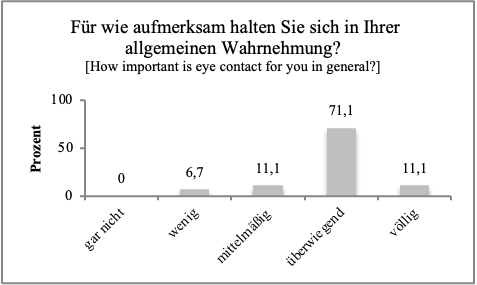

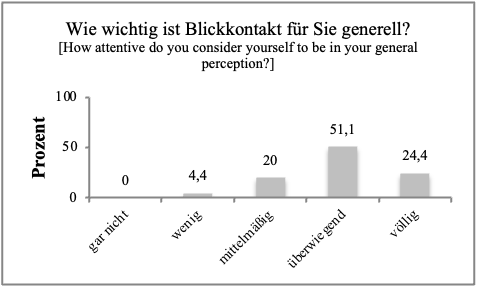

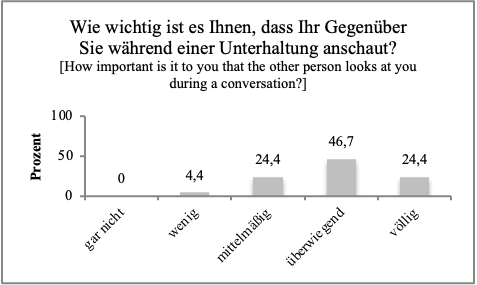

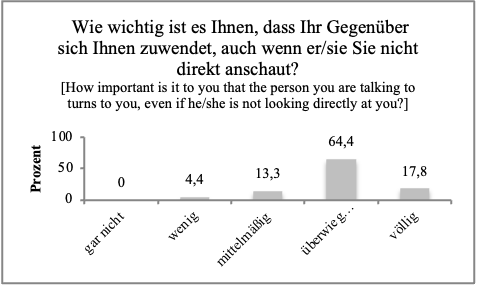

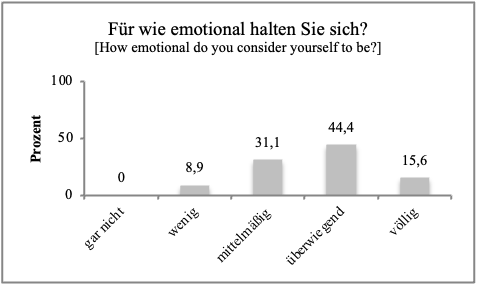


**Supplementary Figure S5.** Experiment 2: Answers to the personal questions.

## Supplementary Tables

**Supplementary Table S1.** Experiment 1: Results of the Hochberg-corrected Shapiro-Wilk tests for mean correct emotion recognition.

| Comparison | Statistic | *df* | *p_corr_* |
| --- | --- | --- | --- |
| Facial expression |  |  |  |
| Anger – fear | 0.95 | 39 | .430 |
| Anger – happiness | 0.66 | 39 | < .001 |
| Anger – neutral | 0.96 | 39 | .473 |
| Anger – sadness | 0.88 | 39 | .007 |
| Fear – happiness | 0.96 | 39 | .473 |
| Fear – neutral | 0.97 | 39 | .473 |
| Fear – sadness | 0.97 | 39 | .473 |
| Happiness – neutral | 0.88 | 39 | .007 |
| Happiness – sadness | 0.82 | 39 | < .001 |
| Neutral – sadness | 0.90 | 39 | .018 |
| Gaze direction |  |  |  |
| Left – centered | 0.95 | 39 | .171 |
| Right – centered | 0.98 | 39 | .630 |
| Left – right | 0.98 | 39 | .630 |
| Head orientation |  |  |  |
| Left – frontal | 0.96 | 39 | .408 |
| Right – frontal | 0.99 | 39 | .908 |
| Left – right | 0.95 | 39 | .264 |

**Supplementary Table S2.** Experiment 1: Results of the Hochberg-corrected Wilcoxon signed-rank tests for correct emotion recognition (percentages given as decimals) (*N*= 39). Pearson's *r* is reported as a measure of effect size.

| Comparison | $\bar{\Delta}$ | *Mdn*  *(x_1_–x_2_)* | 95 % CI | *z* | *p_corr_* | *r* |
| --- | --- | --- | --- | --- | --- | --- |
| Facial expression |  |  |  |  |  |  |
| Anger – fear | 0.30 | 0.25 | [0.22, 0.38] | –4.97 | < .001 | .80 |
| Anger – happiness | –0.06 | –0.03 | [–0.10, –0.02] | –3.93 | < .001 | .63 |
| Anger – neutral | 0.12 | 0.08 | [0.04, 0.19] | –3.08 | .006 | .49 |
| Anger – sadness | 0.06 | 0.03 | [0.01, 0.12] | –2.70 | .014 | .43 |
| Fear – happiness | –0.36 | –0.36 | [–0.44, –0.28] | –5.30 | < .001 | .85 |
| Fear – neutral | –0.18 | –0.22 | [–0.27, –0.10] | –3.56 | < .001 | .57 |
| Fear – sadness | –0.24 | –0.28 | [–0.33, –0.15] | –4.16 | < .001 | .67 |
| Happiness – neutral | 0.18 | 0.11 | [0.12, 0.24] | –4.94 | < .001 | .79 |
| Happiness – sadness | 0.12 | 0.08 | [0.08, 0.17] | –4.94 | < .001 | .79 |
| Neutral – sadness | –0.05 | –0.03 | [–0.12, 0.01] | –1.21 | .227 | .19 |
| Gaze direction |  |  |  |  |  |  |
| Left – centered | –0.04 | –0.05 | [–0.06, –0.02] | –3.45 | .002 | .55 |
| Right – centered | –0.01 | 0.00 | [–0.03, 0.01] | –1.06 | .290 | .17 |
| Left – right | –0.03 | –0.03 | [–0.05, –0.01] | –3.32 | .002 | .53 |
| Head orientation |  |  |  |  |  |  |
| Left – frontal | 0.003 | 0.000 | [–0.01, 0.02] | –0.38 | .901 | .06 |
| Right – frontal | 0.002 | 0.000 | [–0.02, 0.02] | –0.20 | .901 | .03 |
| Left – right | 0.001 | 0.000 | [–0.01, 0.02] | –0.12 | .901 | .02 |

**Supplementary Table S3.** Experiment 1: Results of the univariate rmANOVAs for mean correct emotion recognition separately for each emotion (*N*= 39). ε ̃ gives the value for the correction of the degrees of freedom of the *F*-test according to Greenhouse-Geisser. $\eta_{p}^{2}$ is reported as a measure of effect size.

| Source | *df_source_* | *df_error_* | $\tilde{\varepsilon}$ | *F_corr_* | *p* | $\eta_{p}^{2}$ |
| --- | --- | --- | --- | --- | --- | --- |
| Anger |  |  |  |  |  |  |
| Gaze direction | 2 | 76 | .94 | 1.32 | .274 | .033 |
| Head orientation | 2 | 76 | .95 | 2.43 | .095 | .060 |
| Gaze direction × head orientation^a^ | 4 | 152 | .78 | 2.36 | .073 | .058 |
| Fear |  |  |  |  |  |  |
| Gaze direction | 2 | 76 | .99 | 5.88 | .004 | .134 |
| Head orientation | 2 | 76 | .98 | 0.36 | .702 | .009 |
| Gaze direction × head orientation | 4 | 152 | .94 | 1.04 | .390 | .027 |
| Happiness |  |  |  |  |  |  |
| Gaze direction | 2 | 76 | .98 | 0.14 | .870 | .004 |
| Head orientation | 2 | 76 | .98 | 0.14 | .870 | .004 |
| Gaze direction × head orientation^a^ | 4 | 152 | .73 | 0.64 | .587 | .017 |
| Neutral |  |  |  |  |  |  |
| Gaze direction | 2 | 76 | .89 | 16.29 | < .001 | .300 |
| Head orientation | 2 | 76 | .94 | 2.02 | .140 | .051 |
| Gaze direction × head orientation | 4 | 152 | .89 | 6.22 | < .001 | .141 |
| Sadness |  |  |  |  |  |  |
| Gaze direction | 2 | 76 | .94 | 5.61 | .005 | .129 |
| Head orientation | 2 | 76 | .90 | 1.46 | .238 | .037 |
| Gaze direction × head orientation | 4 | 152 | .88 | 0.14 | .966 | .004 |

^a^*Greenhouse-Geisser corrected due to violation of sphericity.*

**Supplementary Table S4.** Experiment 1: Results of the Hochberg-corrected Shapiro-Wilk tests for mean correct emotion recognition.

| Comparison | Statistic | *df* | *p_corr_* |
| --- | --- | --- | --- |
| Fear  Gaze direction |  |  |  |
| Left – centered | 0.94 | 39 | .083 |
| Right – centered | 0.95 | 39 | .083 |
| Left – right | 0.95 | 39 | .083 |
| Neutral  Gaze direction |  |  |  |
| Left – centered | 0.91 | 39 | .006 |
| Right – centered | 0.91 | 39 | .006 |
| Left – right | 0.87 | 39 | < .001 |
| Sadness  Gaze direction |  |  |  |
| Left – centered | 0.92 | 39 | .008 |
| Right – centered | 0.92 | 39 | .008 |
| Left – right | 0.91 | 39 | .008 |

**Supplementary Table S5.** Experiment 1: Results of the Hochberg-corrected Wilcoxon signed-rank tests for correct emotion recognition (percentages given as decimals) (*N*= 39). Pearson's *r* is reported as a measure of effect size.

| Comparison | $\bar{\Delta}$ | *Mdn*  *(x_1_–x_2_)* | 95 % CI | *z* | *p_corr_* | *r* |
| --- | --- | --- | --- | --- | --- | --- |
| Fear  Gaze direction |  |  |  |  |  |  |
| Left – centered | 0.01 | 0.00 | [–0.04, 0.07] | –0.45 | .654 | .07 |
| Right – centered | 0.09 | 0.00 | [0.03, 0.14] | –2.97 | .009 | .48 |
| Left – right | –0.07 | –0.08 | [–0.13, –0.02] | –2.46 | .028 | .39 |
| Neutral  Gaze direction |  |  |  |  |  |  |
| Left – centered | –0.13 | –0.08 | [–0.19, –0.07] | –3.86 | < .001 | .62 |
| Right – centered | –0.11 | –0.08 | [–0.16, –0.06] | –3.61 | < .001 | .58 |
| Left – right | –0.02 | 0.00 | [–0.06, 0.02] | –1.06 | .288 | .17 |
| Sadness  Gaze direction |  |  |  |  |  |  |
| Left – centered | –0.07 | 0.00 | [–0.12, –0.02] | –2.84 | .015 | .45 |
| Right – centered | –0.03 | 0.00 | [–0.07, 0.01] | –1.65 | .099 | .26 |
| Left – right | –0.04 | 0.00 | [–0.08, 0.00] | –2.14 | .064 | .34 |

**Supplementary Table S6.** Experiment 1: Results of the univariate rmANOVA for mean perceived gaze direction (*N* = 39). ε ̃ gives the value for the correction of the degrees of freedom of the *F*-test according to Greenhouse-Geisser. $\eta_{p}^{2}$ is reported as a measure of effect size.

| Source | *df_source_* | *df_error_* | $\tilde{\varepsilon}$ | *F_corr_* | *p* | $\eta_{p}^{2}$ |
| --- | --- | --- | --- | --- | --- | --- |
| Emotion | 4 | 152 | .86 | 8.19 | < .001 | .177 |
| Gaze direction^a^ | 2 | 76 | .56 | 451.64 | < .001 | .922 |
| Head orientation^a^ | 2 | 76 | .51 | 757.17 | < .001 | .952 |
| Emotion × gaze direction^a^ | 8 | 304 | .63 | 24.27 | < .001 | .390 |
| Emotion × head orientation^a^ | 8 | 304 | .73 | 4.72 | < .001 | .111 |
| Gaze direction × head orientation^a^ | 4 | 152 | .38 | 175.69 | < .001 | .822 |
| Emotion × gaze direction × head orientation^a^ | 16 | 608 | .63 | 2.48 | .007 | .061 |

^a^*Greenhouse-Geisser corrected due to violation of sphericity.*

**Supplementary Table S7.** Experiment 1: Results of the univariate rmANOVAs for mean perceived gaze direction separately for each head orientation (*N* = 39). ε ̃ gives the value for the correction of the degrees of freedom of the *F*-test according to Greenhouse-Geisser. $\eta_{p}^{2}$ is reported as a measure of effect size.

| Source | *df_source_* | *df_error_* | $\tilde{\varepsilon}$ | *F* | *p* | $\eta_{p}^{2}$ |
| --- | --- | --- | --- | --- | --- | --- |
| Left head orientation |  |  |  |  |  |  |
| Gaze direction^a^ | 2 | 76 | .64 | 125.43 | < .001 | .767 |
| Frontal head orientation |  |  |  |  |  |  |
| Gaze direction^a^ | 2 | 76 | .54 | 414.77 | < .001 | .916 |
| Right head orientation |  |  |  |  |  |  |
| Gaze direction^a^ | 2 | 76 | .67 | 123.56 | < .001 | .765 |

^a^*Greenhouse-Geisser corrected due to violation of sphericity.*

**Supplementary Table S8.** Experiment 1: Results of the Hochberg-corrected paired-samples t-tests (two-tailed) for mean perceived gaze direction. Cohen's *d_z_* is reported as a measure of effect size.

| Comparison | $\bar{\Delta}$ | *t* | *df* | *p_corr_* | *d_z_* |
| --- | --- | --- | --- | --- | --- |
| Left head orientation  Gaze direction |  |  |  |  |  |
| Left – centered | –7.98 | –8.91 | 38 | < .001 | –1.43 |
| Right – centered | 7.65 | 11.57 | 38 | < .001 | 1.85 |
| Left – right | –15.63 | –12.05 | 38 | < .001 | –1.93 |
| Frontal head orientation  Gaze direction |  |  |  |  |  |
| Left – centered | –32.17 | –19.97 | 38 | < .001 | –3.20 |
| Right – centered | 25.90 | 19.32 | 38 | < .001 | 3.09 |
| Left – right | –58.07 | –20.72 | 38 | < .001 | –3.32 |
| Right head orientation  Gaze direction |  |  |  |  |  |
| Left – centered | –11.96 | –12.02 | 38 | < .001 | –1.92 |
| Right – centered | 5.81 | 6.72 | 38 | < .001 | 1.08 |
| Left – right | –17.77 | –11.85 | 38 | < .001 | –1.90 |

**Supplementary Table S9.** Experiment 1: Results of the univariate rmANOVA for mean perceived head orientation (*N*= 39). ε ̃ gives the value for the correction of the degrees of freedom of the *F*-test according to Greenhouse-Geisser. $\eta_{p}^{2}$ is reported as a measure of effect size.

| Source | *df_source_* | *df_error_* | $\tilde{\varepsilon}$ | *F_corr_* | *p* | $\eta_{p}^{2}$ |
| --- | --- | --- | --- | --- | --- | --- |
| Emotion | 4 | 152 | .90 | 3.15 | .016 | .076 |
| Gaze direction^a^ | 2 | 76 | .64 | 8.67 | .003 | .186 |
| Head orientation^a^ | 2 | 76 | .52 | 1090.28 | < .001 | .966 |
| Emotion × gaze direction | 8 | 304 | .78 | 3.68 | .001 | .088 |
| Emotion × head orientation | 8 | 304 | .78 | 7.45 | < .001 | .164 |
| Gaze direction × head orientation^a^ | 4 | 152 | .72 | 12.50 | < .001 | .248 |
| Emotion × gaze direction × head orientation^a^ | 16 | 608 | .64 | 1.29 | .233 | .033 |

^a^*Greenhouse-Geisser corrected due to violation of sphericity.*

**Supplementary Table S10.** Experiment 1: Results of the univariate rmANOVAs for mean perceived head orientation separately for each head orientation (*N*= 39). ε ̃ gives the value for the correction of the degrees of freedom of the *F*-test according to Greenhouse-Geisser. $\eta_{p}^{2}$ is reported as a measure of effect size.

| Source | *df_source_* | *df_error_* | $\tilde{\varepsilon}$ | *F_corr_* | *p* | $\eta_{p}^{2}$ |
| --- | --- | --- | --- | --- | --- | --- |
| Left head orientation |  |  |  |  |  |  |
| Gaze direction^a^ | 2 | 76 | .87 | 0.93 | .389 | .024 |
| Frontal head orientation |  |  |  |  |  |  |
| Gaze direction^a^ | 2 | 76 | .55 | 28.86 | < .001 | .432 |
| Right head orientation |  |  |  |  |  |  |
| Gaze direction^a^ | 2 | 76 | .71 | 3.50 | .052 | .084 |

^a^*Greenhouse-Geisser corrected due to violation of sphericity.*

**Supplementary Table S11.** Experiment 1: Results of the Hochberg-corrected paired-samples t-tests (two-tailed) for mean perceived head orientation. Cohen's *d_z_* is reported as a measure of effect size.

| Comparison | $\bar{\Delta}$ | *t* | *df* | *p_corr_* | *d_z_* |
| --- | --- | --- | --- | --- | --- |
| Frontal head orientation  Gaze direction |  |  |  |  |  |
| Left – centered | –2.10 | –5.16 | 38 | < .001 | –0.83 |
| Right – centered | 2.52 | 5.11 | 38 | < .001 | 0.82 |
| Left – right | –4.62 | –5.51 | 38 | < .001 | –0.88 |

**Supplementary Table S12.** Experiment 2: Results of the Hochberg-corrected Shapiro-Wilk tests for mean correct emotion recognition.

| Comparison | Statistic | *df* | *p_corr_* |
| --- | --- | --- | --- |
| Facial expression |  |  |  |
| Anger – fear | 0.96 | 45 | .745 |
| Anger – happiness | 0.92 | 45 | .054 |
| Anger – neutral | 0.98 | 45 | .745 |
| Anger – sadness | 0.98 | 45 | .745 |
| Fear – happiness | 0.98 | 45 | .745 |
| Fear – neutral | 0.98 | 45 | .745 |
| Fear – sadness | 0.97 | 45 | .745 |
| Happiness – neutral | 0.92 | 45 | .040 |
| Happiness – sadness | 0.98 | 45 | .745 |
| Neutral – sadness | 0.98 | 45 | .745 |
| Gaze direction |  |  |  |
| Left – centered | 0.98 | 45 | .521 |
| Right – centered | 0.96 | 45 | .270 |
| Left – right | 0.95 | 45 | .213 |
| Head orientation |  |  |  |
| Left – frontal | 0.97 | 45 | .607 |
| Right – frontal | 0.96 | 45 | .330 |
| Left – right | 0.98 | 45 | .607 |

**Supplementary Table S13.** Experiment 2: Results of the Hochberg-corrected Wilcoxon signed-rank tests for correct emotion recognition (percentages given as decimals) (*N*= 45). Pearson's *r* is reported as a measure of effect size.

| Comparison | $\bar{\Delta}$ | *Mdn*  *(x_1_–x_2_)* | 95 % CI | *z* | *p_corr_* | *r* |
| --- | --- | --- | --- | --- | --- | --- |
| Facial expression |  |  |  |  |  |  |
| Anger – fear | 0.20 | 0.19 | [0.13, 0.26] | –4.74 | < .001 | .71 |
| Anger – happiness | –0.17 | –0.17 | [–0.22, –0.12] | –5.36 | < .001 | .80 |
| Anger – neutral | –0.07 | –0.06 | [–0.13, –0.02] | –2.59 | .020 | .39 |
| Anger – sadness | 0.23 | 0.19 | [0.15, 0.32] | –4.47 | < .001 | .67 |
| Fear – happiness | –0.37 | –0.36 | [–0.42, –0.31] | –5.84 | < .001 | .87 |
| Fear – neutral | –0.27 | –0.28 | [–0.34, –0.20] | –5.29 | < .001 | .79 |
| Fear – sadness | 0.04 | 0.00 | [–0.04, 0.11] | –0.77 | .444 | .11 |
| Happiness – neutral | 0.10 | 0.08 | [0.06, 0.13] | –4.44 | < .001 | .66 |
| Happiness – sadness | 0.40 | 0.39 | [0.34, 0.47] | –5.78 | < .001 | .86 |
| Neutral – sadness | 0.31 | 0.28 | [0.24, 0.38] | –5.48 | < .001 | .82 |
| Gaze direction |  |  |  |  |  |  |
| Left – centered | –0.06 | –0.05 | [–0.08, –0.04] | –4.80 | < .001 | .72 |
| Right – centered | 0.01 | 0.00 | [–0.01, 0.03] | –0.25 | .803 | .04 |
| Left – right | –0.07 | –0.05 | [–0.08, –0.05] | –5.45 | < .001 | .81 |
| Head orientation |  |  |  |  |  |  |
| Left – frontal | 0.019 | 0.02 | [0.00, 0.04] | –2.06 | .117 | .31 |
| Right – frontal | 0.004 | 0.00 | [–0.01, 0.02] | –0.39 | .693 | .06 |
| Left – right | 0.015 | 0.02 | [0.00, 0.03] | –1.85 | .130 | .28 |

**Supplementary Table S14.** Experiment 2: Results of the mixed ANCOVA for mean correct emotion recognition (*N* = 84). ε ̃ gives the value for the correction of the degrees of freedom of the *F*-test according to Greenhouse-Geisser. $\eta_{p}^{2}$ is reported as a measure of effect size.

| Source | *df_source_* | *df_error_* | $\tilde{\varepsilon}$ | *F_corr_* | *p* | $\eta_{p}^{2}$ |
| --- | --- | --- | --- | --- | --- | --- |
| *Gender* | 1 | 81 | – | 0.01 | .917 | .000 |
| Mask | 1 | 81 | – | 25.35 | < .001 | .238 |
| Emotion^a^ | 4 | 324 | .82 | 7.97 | < .001 | .090 |
| Emotion × mask^a^ | 4 | 324 | .82 | 18.08 | < .001 | .182 |
| Gaze direction | 2 | 162 | .96 | 4.16 | .017 | .049 |
| Gaze direction × mask | 2 | 162 | .96 | 3.02 | .052 | .036 |
| Head orientation | 2 | 162 | .99 | 1.27 | .285 | .015 |
| Head orientation × mask | 2 | 162 | .99 | 1.34 | .264 | .016 |
| Emotion × gaze direction ^a^ | 8 | 648 | .72 | 3.49 | .003 | .041 |
| Emotion × gaze direction × mask^a^ | 8 | 648 | .72 | 6.82 | < .001 | .078 |
| Emotion × head orientation^a^ | 8 | 648 | .76 | 0.43 | .860 | .005 |
| Emotion × head orientation × mask^a^ | 8 | 648 | .76 | 1.45 | .192 | .018 |
| Gaze direction × head orientation^a^ | 4 | 324 | .88 | 0.57 | .661 | .007 |
| Gaze direction × head orientation × mask^a^ | 4 | 324 | .88 | 0.77 | .528 | .009 |
| Emotion × gaze direction × head orientation^a^ | 16 | 1296 | .69 | 0.95 | .489 | .012 |
| Emotion × gaze direction × head orientation × mask^a^ | 16 | 1296 | .69 | 1.02 | .423 | .012 |

^a^*Greenhouse-Geisser corrected due to violation of sphericity.*

**Supplementary Table S15.** Experiment 2: Results of the univariate rmANOVAs for mean correct emotion recognition separately for each emotion (*N* = 45). ε ̃ gives the value for the correction of the degrees of freedom of the *F*-test according to Greenhouse-Geisser. $\eta_{p}^{2}$ is reported as a measure of effect size.

| Source | *df_source_* | *df_error_* | $\tilde{\varepsilon}$ | *F_corr_* | *p* | $\eta_{p}^{2}$ |
| --- | --- | --- | --- | --- | --- | --- |
| Anger |  |  |  |  |  |  |
| Gaze direction | 2 | 88 | .93 | 29.74 | < .001 | .403 |
| Head orientation | 2 | 88 | .95 | 4.18 | .019 | .087 |
| Gaze direction × head orientation^a^ | 4 | 176 | .74 | 1.43 | .237 | .031 |
| Fear |  |  |  |  |  |  |
| Gaze direction^a^ | 2 | 88 | .85 | 31.82 | < .001 | .420 |
| Head orientation | 2 | 88 | .97 | 0.26 | .774 | .006 |
| Gaze direction × head orientation | 4 | 176 | .88 | 0.54 | .706 | .012 |
| Happiness |  |  |  |  |  |  |
| Gaze direction | 2 | 88 | .99 | 2.76 | .069 | .059 |
| Head orientation | 2 | 88 | .94 | 3.22 | .045 | .068 |
| Gaze direction × head orientation | 4 | 176 | .88 | 3.38 | .011 | .071 |
| Neutral |  |  |  |  |  |  |
| Gaze direction^a^ | 2 | 88 | .81 | 21.54 | < .001 | .329 |
| Head orientation^a^ | 2 | 88 | .79 | 7.87 | .002 | .152 |
| Gaze direction × head orientation^a^ | 4 | 176 | .91 | 4.10 | .003 | .085 |
| Sadness |  |  |  |  |  |  |
| Gaze direction | 2 | 88 | 1 | 22.32 | < .001 | .337 |
| Head orientation | 2 | 88 | .96 | 0.68 | .508 | .015 |
| Gaze direction × head orientation | 4 | 176 | .94 | 1.31 | .270 | .029 |

^a^*Greenhouse-Geisser corrected due to violation of sphericity.*

**Supplementary Table S16.** Experiment 2: Results of the Hochberg-corrected Shapiro-Wilk tests for mean correct emotion recognition.

| Comparison | Statistic | *df* | *p_corr_* |
| --- | --- | --- | --- |
| Anger  Gaze direction |  |  |  |
| Left – centered | 0.95 | 45 | .076 |
| Right – centered | 0.96 | 45 | .090 |
| Left – right | 0.94 | 45 | .060 |
| Fear  Gaze direction |  |  |  |
| Left – centered | 0.96 | 45 | .267 |
| Right – centered | 0.97 | 45 | .267 |
| Left – right | 0.96 | 45 | .267 |
| Neutral  Gaze direction |  |  |  |
| Left – centered | 0.89 | 45 | .002 |
| Right – centered | 0.86 | 45 | < .001 |
| Left – right | 0.93 | 45 | .014 |
| Sadness  Gaze direction |  |  |  |
| Left – centered | 0.97 | 45 | .218 |
| Right – centered | 0.95 | 45 | .141 |
| Left – right | 0.96 | 45 | .218 |

**Supplementary Table S17.** Experiment 2: Results of the Hochberg-corrected Wilcoxon signed-rank tests for correct emotion recognition (percentages given as decimals) (*N*= 45). Pearson's *r* is reported as a measure of effect size.

| Comparison | $\bar{\Delta}$ | *Mdn*  *(x_1_–x_2_)* | 95 % CI | *z* | *p_corr_* | *r* |
| --- | --- | --- | --- | --- | --- | --- |
| Anger  Gaze direction |  |  |  |  |  |  |
| Left – centered | –0.16 | –0.17 | [–0.20, –0.11] | –4.96 | < .001 | .74 |
| Right – centered | –0.02 | 0.00 | [–0.06, 0.02] | –0.91 | .361 | .14 |
| Left – right | –0.14 | –0.08 | [–0.18, –0.09] | –4.64 | < .001 | .69 |
| Fear  Gaze direction |  |  |  |  |  |  |
| Left – centered | 0.15 | 0.17 | [0.09, 0.20] | –4.08 | < .001 | .61 |
| Right – centered | 0.20 | 0.17 | [0.14, 0.25] | –4.97 | < .001 | .74 |
| Left – right | –0.05 | –0.08 | [–0.09, –0.01] | –2.45 | .014 | .37 |
| Neutral  Gaze direction |  |  |  |  |  |  |
| Left – centered | –0.12 | ­–0.08 | [–0.16, –0.08] | –4.55 | < .001 | .68 |
| Right – centered | –0.07 | ­–0.08 | [–0.10, –0.04] | –4.15 | < .001 | .62 |
| Left – right | –0.05 | 0.00 | [–0.10, –0.01] | –2.43 | .015 | .36 |
| Sadness  Gaze direction |  |  |  |  |  |  |
| Left – centered | –0.17 | ­–0.17 | [–0.22, –0.12] | –4.70 | < .001 | .70 |
| Right – centered | –0.06 | ­–0.08 | [–0.11, –0.01] | –2.01 | .045 | .30 |
| Left – right | –0.11 | –0.08 | [–0.16, –0.06] | –3.61 | < .001 | .54 |

**Supplementary Table S18.** Experiment 2: Results of the Hochberg-corrected Shapiro-Wilk tests for mean correct emotion recognition.

| Comparison | Statistic | *df* | *p_corr_* |
| --- | --- | --- | --- |
| Anger  Head orientation |  |  |  |
| Left – frontal | 0.94 | 45 | .087 |
| Right – frontal | 0.97 | 45 | .225 |
| Left – right | 0.96 | 45 | .225 |
| Happiness  Head orientation |  |  |  |
| Left – frontal | 0.81 | 45 | < .001 |
| Right – frontal | 0.85 | 45 | < .001 |
| Left – right | 0.74 | 45 | < .001 |
| Neutral  Head orientation |  |  |  |
| Left – frontal | 0.87 | 45 | < .001 |
| Right – frontal | 0.93 | 45 | .009 |
| Left – right | 0.84 | 45 | < .001 |

**Supplementary Table S19.** Experiment 2: Results of the Hochberg-corrected Wilcoxon signed-rank tests for correct emotion recognition (percentages given as decimals) (*N*= 45). Pearson's *r* is reported as a measure of effect size.

| Comparison | $\bar{\Delta}$ | *Mdn*  *(x_1_–x_2_)* | 95 % CI | *z* | *p_corr_* | *r* |
| --- | --- | --- | --- | --- | --- | --- |
| Anger  Head orientation |  |  |  |  |  |  |
| Left – frontal | –0.04 | 0.00 | [–0.07, 0.00] | –1.78 | .150 | .27 |
| Right – frontal | –0.06 | –0.08 | [–0.11, –0.01] | –2.36 | .054 | .35 |
| Left – right | 0.02 | 0.08 | [–0.02, 0.06] | –1.14 | .256 | .17 |
| Happiness  Head orientation |  |  |  |  |  |  |
| Left – frontal | 0.02 | 0.00 | [0.01, 0.04] | –2.60 | .027 | .39 |
| Right – frontal | 0.01 | 0.00 | [–0.01, 0.04] | –1.31 | .308 | .20 |
| Left – right | 0.01 | 0.00 | [–0.01, 0.03] | –1.02 | .308 | .15 |
| Neutral  Head orientation |  |  |  |  |  |  |
| Left – frontal | 0.07 | 0.00 | [0.03, 0.11] | –3.52 | < .001 | .52 |
| Right – frontal | 0.05 | 0.00 | [0.01, 0.09] | –2.10 | .072 | .31 |
| Left – right | 0.02 | 0.00 | [–0.01, 0.05] | –1.41 | .160 | .21 |

**Supplementary Table S20.** Experiment 2: Results of the mixed ANCOVA for mean perceived gaze direction (*N* = 84). ε ̃ gives the value for the correction of the degrees of freedom of the *F*-test according to Greenhouse-Geisser. $\eta_{p}^{2}$ is reported as a measure of effect size.

| Source | *df_source_* | *df_error_* | $\tilde{\varepsilon}$ | *F_corr_* | *p* | $\eta_{p}^{2}$ |
| --- | --- | --- | --- | --- | --- | --- |
| *Gender* | 1 | 81 | – | 0.10 | .758 | .001 |
| Mask | 1 | 81 | – | 6.58 | .012 | .075 |
| Emotion^a^ | 4 | 324 | .89 | 3.97 | .005 | .047 |
| Emotion × mask^a^ | 4 | 324 | .89 | 0.52 | .702 | .006 |
| Gaze direction^a^ | 2 | 162 | .55 | 80.54 | < .001 | .499 |
| Gaze direction × mask^a^ | 2 | 162 | .55 | 0.25 | .639 | .003 |
| Head orientation^a^ | 2 | 162 | .52 | 176.83 | < .001 | .686 |
| Head orientation × mask^a^ | 2 | 162 | .52 | 1.83 | .180 | .022 |
| Emotion × gaze direction ^a^ | 8 | 648 | .76 | 4.91 | < .001 | .057 |
| Emotion × gaze direction × mask^a^ | 8 | 648 | .76 | 0.64 | .701 | .008 |
| Emotion × head orientation^a^ | 8 | 648 | .78 | 3.45 | .002 | .041 |
| Emotion × head orientation × mask^a^ | 8 | 648 | .78 | 1.56 | .156 | .019 |
| Gaze direction × head orientation^a^ | 4 | 324 | .37 | 46.85 | < .001 | .366 |
| Gaze direction × head orientation × mask^a^ | 4 | 324 | .37 | 1.71 | .192 | .021 |
| Emotion × gaze direction × head orientation^a^ | 16 | 1296 | .77 | 2.65 | .002 | .032 |
| Emotion × gaze direction × head orientation × mask^a^ | 16 | 1296 | .77 | 1.33 | .192 | .016 |

^a^*Greenhouse-Geisser corrected due to violation of sphericity.*

**Supplementary Table S21.** Experiment 2: Results of the univariate rmANOVAs for mean perceived gaze direction (*N* = 45). ε ̃ gives the value for the correction of the degrees of freedom of the *F*-test according to Greenhouse-Geisser. $\eta_{p}^{2}$ is reported as a measure of effect size.

| Source | *df_source_* | *df_error_*) | $\tilde{\varepsilon}$ | *F_corr_* | *p* | $\eta_{p}^{2}$ |
| --- | --- | --- | --- | --- | --- | --- |
| Emotion | 4 | 176 | .84 | 23.90 | < .001 | .352 |
| Gaze direction^a^ | 2 | 88 | .53 | 395.10 | < .001 | .900 |
| Head orientation^a^ | 2 | 88 | .53 | 1105.90 | < .001 | .962 |
| Emotion × gaze direction^a^ | 8 | 352 | .74 | 27.81 | < .001 | .387 |
| Emotion × head orientation^a^ | 8 | 352 | .72 | 6.29 | < .001 | .125 |
| Gaze direction × head orientation^a^ | 4 | 176 | .36 | 151.27 | < .001 | .775 |
| Emotion × gaze direction × head orientation^a^ | 16 | 704 | .63 | 2.70 | .003 | .058 |

^a^*Greenhouse-Geisser corrected due to violation of sphericity.*

**Supplementary Table S22.** Experiment 2: Results of the univariate rmANOVAs for mean perceived gaze direction separately for each head orientation (*N* = 45). ε ̃ gives the value for the correction of the degrees of freedom of the *F*-test according to Greenhouse-Geisser. $\eta_{p}^{2}$ is reported as a measure of effect size.

| Source | *df_source_* | *df_error_* | $\tilde{\varepsilon}$ | *F_corr_* | *p* | $\eta_{p}^{2}$ |
| --- | --- | --- | --- | --- | --- | --- |
| Left head orientation |  |  |  |  |  |  |
| Gaze direction^a^ | 2 | 88 | .66 | 142.62 | < .001 | .764 |
| Frontal head orientation |  |  |  |  |  |  |
| Gaze direction^a^ | 2 | 88 | .54 | 388.76 | < .001 | .898 |
| Right head orientation |  |  |  |  |  |  |
| Gaze direction^a^ | 2 | 88 | .60 | 117.77 | < .001 | .728 |

^a^*Greenhouse-Geisser corrected due to violation of sphericity.*

**Supplementary Table S23.** Experiment 2: Results of the Hochberg-corrected paired-samples t-tests (two-tailed) for mean perceived gaze direction. Cohen's *d_z_* is reported as a measure of effect size.

| Comparison | $\bar{\Delta}$ | *t* | *df* | *p_corr_* | *d_z_* |
| --- | --- | --- | --- | --- | --- |
| Left head orientation  Gaze direction |  |  |  |  |  |
| Left – centered | –8.72 | –10.40 | 44 | < .001 | –1.55 |
| Right – centered | 9.05 | 10.64 | 44 | < .001 | 1.59 |
| Left – right | –17.77 | –12.91 | 44 | < .001 | –1.92 |
| Frontal head orientation  Gaze direction |  |  |  |  |  |
| Left – centered | –28.39 | –21.46 | 44 | < .001 | –3.20 |
| Right – centered | 25.21 | 16.82 | 44 | < .001 | 2.51 |
| Left – right | –53.61 | –20.11 | 44 | < .001 | –3.00 |
| Right head orientation  Gaze direction |  |  |  |  |  |
| Left – centered | –12.52 | –10.83 | 44 | < .001 | –1.62 |
| Right – centered | 5.29 | 7.71 | 44 | < .001 | 1.15 |
| Left – right | –17.81 | –11.37 | 44 | < .001 | –1.69 |

**Supplementary Table S24.** Experiment 2: Results of the mixed ANCOVA for mean perceived head orientation (*N* = 84). ε ̃ gives the value for the correction of the degrees of freedom of the *F*-test according to Greenhouse-Geisser. $\eta_{p}^{2}$ is reported as a measure of effect size.

| Source | *df_source_* | *df_error_* | $\tilde{\varepsilon}$ | *F_corr_* | *p* | $\eta_{p}^{2}$ |
| --- | --- | --- | --- | --- | --- | --- |
| *Gender* | 1 | 81 | – | 0.00 | .995 | .000 |
| Mask | 1 | 81 | – | 1.93 | .169 | .023 |
| Emotion | 4 | 324 | .94 | 0.98 | .421 | .012 |
| Emotion × mask | 4 | 324 | .94 | 1.42 | .226 | .017 |
| Gaze direction^a^ | 2 | 162 | .58 | 0.16 | .726 | .002 |
| Gaze direction × mask^a^ | 2 | 162 | .58 | 0.42 | .549 | .005 |
| Head orientation^a^ | 2 | 162 | .52 | 227.99 | < .001 | .738 |
| Head orientation × mask^a^ | 2 | 162 | .52 | 1.28 | .264 | .016 |
| Emotion × gaze direction ^a^ | 8 | 648 | .86 | 0.85 | .546 | .001 |
| Emotion × gaze direction × mask^a^ | 8 | 648 | .86 | 2.04 | .049 | .025 |
| Emotion × head orientation^a^ | 8 | 648 | .83 | 1.96 | .063 | .024 |
| Emotion × head orientation × mask^a^ | 8 | 648 | .83 | 1.10 | .364 | .013 |
| Gaze direction × head orientation^a^ | 4 | 324 | .59 | 1.51 | .221 | .018 |
| Gaze direction × head orientation × mask^a^ | 4 | 324 | .59 | 0.69 | .526 | .008 |
| Emotion × gaze direction × head orientation^a^ | 16 | 1296 | .76 | 1.17 | .300 | .014 |
| Emotion × gaze direction × head orientation × mask^a^ | 16 | 1296 | .76 | 1.69 | .062 | .020 |

^a^*Greenhouse-Geisser corrected due to violation of sphericity.*

**Supplementary Table S25.** Experiment 2: Results of the univariate rmANOVAs for mean perceived head orientation (*N* = 45). ε ̃ gives the value for the correction of the degrees of freedom of the *F*-test according to Greenhouse-Geisser. $\eta_{p}^{2}$ is reported as a measure of effect size.

| Source | *df_source_* | *df_error_* | $\tilde{\varepsilon}$ | *F_corr_* | *p* | $\eta_{p}^{2}$ |
| --- | --- | --- | --- | --- | --- | --- |
| Emotion | 4 | 176 | .90 | 1.79 | .134 | .039 |
| Gaze direction^a^ | 2 | 88 | .56 | 6.42 | .012 | .127 |
| Head orientation^a^ | 2 | 88 | .52 | 1190.51 | < .001 | .964 |
| Emotion × gaze direction^a^ | 8 | 352 | .78 | 2.62 | .008 | .056 |
| Emotion × head orientation^a^ | 8 | 352 | .77 | 2.99 | .007 | .064 |
| Gaze direction × head orientation^a^ | 4 | 176 | .51 | 3.93 | .023 | .082 |
| Emotion × gaze direction × head orientation^a^ | 16 | 704 | .67 | 1.67 | .080 | .037 |

^a^*Greenhouse-Geisser corrected due to violation of sphericity.*

**Supplementary Table S26.** Experiment 2: Results of the univariate rmANOVAs for mean perceived head orientation separately for each head orientation (*N* = 45). ε ̃ gives the value for the correction of the degrees of freedom of the *F*-test according to Greenhouse-Geisser. $\eta_{p}^{2}$ is reported as a measure of effect size.

| Source | *df_source_* | *df_error_* | $\tilde{\varepsilon}$ | *F_corr_* | *p* | $\eta_{p}^{2}$ |
| --- | --- | --- | --- | --- | --- | --- |
| Left head orientation |  |  |  |  |  |  |
| Gaze direction^a^ | 2 | 88 | .76 | 3.93 | .035 | .082 |
| Frontal head orientation |  |  |  |  |  |  |
| Gaze direction^a^ | 2 | 88 | .55 | 7.74 | .006 | .150 |
| Right head orientation |  |  |  |  |  |  |
| Gaze direction^a^ | 2 | 88 | .74 | 1.47 | .237 | .032 |

^a^*Greenhouse-Geisser corrected due to violation of sphericity.*

**Supplementary Table S27.** Experiment 2: Results of the Hochberg-corrected paired-samples t-tests (two-tailed) for mean perceived gaze direction. Cohen's *d_z_* is reported as a measure of effect size.

| Comparison | $\bar{\Delta}$ | *t* | *df* | *p_corr_* | *d_z_* |
| --- | --- | --- | --- | --- | --- |
| Left head orientation  Gaze direction |  |  |  |  |  |
| Left – centered | –1.26 | –2.01 | 44 | .102 | –0.30 |
| Right – centered | 0.77 | 1.26 | 44 | .215 | 0.19 |
| Left – right | –2.02 | –2.22 | 44 | .093 | –0.33 |
| Frontal head orientation  Gaze direction |  |  |  |  |  |
| Left – centered | –2.06 | –2.28 | 44 | .027 | –0.34 |
| Right – centered | 2.76 | 3.04 | 44 | .012 | 0.45 |
| Left – right | –4.82 | –2.84 | 44 | .014 |  |

**Supplementary Table S28.** Experiment 1: The data for emotion recognition performance (given in percent correct).

| Stimulus | N | *M* | *SE* |
| --- | --- | --- | --- |
| E_AL135 | 39 | 94.2 | 2.3 |
| E_AF135 | 39 | 95.5 | 1.8 |
| E_AR135 | 39 | 94.9 | 2.1 |
| E_AL90 | 39 | 89.1 | 3.3 |
| E_AF90 | 39 | 96.2 | 2.2 |
| E_AR90 | 39 | 91.7 | 2.3 |
| E_AL45 | 39 | 93.0 | 2.0 |
| E_AF45 | 39 | 90.4 | 2.8 |
| E_AR45 | 39 | 95.5 | 2.0 |
| E_FL135 | 39 | 60.9 | 3.8 |
| E_FF135 | 39 | 56.4 | 5.8 |
| E_FR135 | 39 | 69.9 | 4.5 |
| E_FL90 | 39 | 64.1 | 4.8 |
| E_FF90 | 39 | 59.6 | 4.6 |
| E_FR90 | 39 | 69.2 | 4.6 |
| E_FL45 | 39 | 59.6 | 4.3 |
| E_FF45 | 39 | 64.1 | 5.3 |
| E_FR45 | 39 | 67.3 | 5.4 |
| E_HL135 | 39 | 100.0 | 0.0 |
| E_HF135 | 39 | 98.7 | 0.9 |
| E_HR135 | 39 | 99.4 | 0.6 |
| E_HL90 | 39 | 99.4 | 0.6 |
| E_HF90 | 39 | 100.0 | 0.0 |
| E_HR90 | 39 | 99.4 | 0.6 |
| E_HL45 | 39 | 99.4 | 0.6 |
| E_HF45 | 39 | 99.4 | 0.6 |
| E_HR45 | 39 | 99.4 | 0.6 |
| E_NL135 | 39 | 82.1 | 3.9 |
| E_NF135 | 39 | 85.9 | 3.0 |
| E_NR135 | 39 | 80.8 | 4.2 |
| E_NL90 | 39 | 73.1 | 4.6 |
| E_NF90 | 39 | 93.6 | 2.2 |
| E_NR90 | 39 | 71.2 | 4.7 |
| E_NL45 | 39 | 75.0 | 4.6 |
| E_NF45 | 39 | 89.7 | 2.6 |
| E_NR45 | 39 | 85.3 | 4.1 |
| E_SL135 | 39 | 83.3 | 3.6 |
| E_SF135 | 39 | 89.1 | 2.7 |
| E_SR135 | 39 | 87.2 | 3.2 |
| E_SL90 | 39 | 84.6 | 2.8 |
| E_SF90 | 39 | 93.0 | 2.2 |
| E_SR90 | 39 | 89.7 | 2.9 |
| E_SL45 | 39 | 82.7 | 3.5 |
| E_SF45 | 39 | 89.7 | 2.9 |
| E_SR45 | 39 | 85.9 | 3.8 |

facial expression: A = anger, F = fear, H = happiness, N = neutral, S = sadness

gaze direction: L = left, F = frontal, R = right

head orientation: 135 = left, 90 = frontal, 45 = right

**Supplementary Table S29.** Experiment 1: The data for perceived gaze direction (given in degrees).

| Stimulus | N | *M* | *SE* |
| --- | --- | --- | --- |
| E_AL135 | 39 | –51.83 | 2.09 |
| E_AF135 | 39 | –45.19 | 1.79 |
| E_AR135 | 39 | –38.88 | 1.98 |
| E_AL90 | 39 | –32.08 | 1.81 |
| E_AF90 | 39 | –0.29 | 0.28 |
| E_AR90 | 39 | 24.39 | 1.30 |
| E_AL45 | 39 | 36.44 | 1.91 |
| E_AF45 | 39 | 47.02 | 2.08 |
| E_AR45 | 39 | 51.57 | 2.27 |
| E_FL135 | 39 | –58.53 | 2.17 |
| E_FF135 | 39 | –45.35 | 1.76 |
| E_FR135 | 39 | –36.15 | 1.79 |
| E_FL90 | 39 | –36.70 | 1.87 |
| E_FF90 | 39 | –0.48 | 0.18 |
| E_FR90 | 39 | 26.96 | 1.45 |
| E_FL45 | 39 | 26.51 | 1.50 |
| E_FF45 | 39 | 47.12 | 1.70 |
| E_FR45 | 39 | 54.94 | 2.38 |
| E_HL135 | 39 | –50.61 | 2.00 |
| E_HF135 | 39 | –44.04 | 1.61 |
| E_HR135 | 39 | –36.83 | 1.89 |
| E_HL90 | 39 | –32.47 | 1.67 |
| E_HF90 | 39 | –0.13 | 0.21 |
| E_HR90 | 39 | 26.63 | 1.59 |
| E_HL45 | 39 | 34.36 | 2.16 |
| E_HF45 | 39 | 45.13 | 1.84 |
| E_HR45 | 39 | 50.35 | 1.94 |
| E_NL135 | 39 | –51.73 | 1.88 |
| E_NF135 | 39 | –43.81 | 1.88 |
| E_NR135 | 39 | –36.57 | 1.79 |
| E_NL90 | 39 | –29.20 | 1.65 |
| E_NF90 | 39 | –0.13 | 0.20 |
| E_NR90 | 39 | 25.38 | 1.62 |
| E_NL45 | 39 | 36.70 | 2.03 |
| E_NF45 | 39 | 44.13 | 1.62 |
| E_NR45 | 39 | 49.42 | 1.81 |
| E_SL135 | 39 | –49.33 | 1.93 |
| E_SF135 | 39 | –43.72 | 1.46 |
| E_SR135 | 39 | –35.45 | 1.65 |
| E_SL90 | 39 | –31.38 | 1.62 |
| E_SF90 | 39 | 0.06 | 0.27 |
| E_SR90 | 39 | 25.16 | 1.40 |
| E_SL45 | 39 | 34.26 | 1.72 |
| E_SF45 | 39 | 44.65 | 1.85 |
| E_SR45 | 39 | 50.83 | 2.06 |

facial expression: A = anger, F = fear, H = happiness, N = neutral, S = sadness

gaze direction: L = left, F = frontal, R = right

head orientation: 135 = left, 90 = frontal, 45 = right

**Supplementary Table S30.** Experiment 1: The data for perceived head orientation (given in degrees).

| Stimulus | N | *M* | *SE* |
| --- | --- | --- | --- |
| E_AL135 | 39 | –45.19 | 1.62 |
| E_AF135 | 39 | –44.81 | 1.55 |
| E_AR135 | 39 | –45.16 | 1.54 |
| E_AL90 | 39 | –2.37 | 0.57 |
| E_AF90 | 39 | –0.32 | 0.24 |
| E_AR90 | 39 | 2.82 | 0.68 |
| E_AL45 | 39 | 47.66 | 1.68 |
| E_AF45 | 39 | 48.08 | 1.97 |
| E_AR45 | 39 | 48.17 | 1.70 |
| E_FL135 | 39 | –46.83 | 1.69 |
| E_FF135 | 39 | –45.19 | 1.55 |
| E_FR135 | 39 | –45.10 | 1.66 |
| E_FL90 | 39 | –2.34 | 0.79 |
| E_FF90 | 39 | –0.45 | 0.23 |
| E_FR90 | 39 | 3.78 | 0.87 |
| E_FL45 | 39 | 51.25 | 1.65 |
| E_FF45 | 39 | 47.60 | 1.61 |
| E_FR45 | 39 | 49.97 | 1.66 |
| E_HL135 | 39 | –43.11 | 1.41 |
| E_HF135 | 39 | –44.84 | 1.49 |
| E_HR135 | 39 | –43.59 | 1.55 |
| E_HL90 | 39 | –1.09 | 0.48 |
| E_HF90 | 39 | –0.16 | 0.15 |
| E_HR90 | 39 | 1.96 | 0.63 |
| E_HL45 | 39 | 49.33 | 1.63 |
| E_HF45 | 39 | 45.87 | 1.49 |
| E_HR45 | 39 | 47.34 | 1.36 |
| E_NL135 | 39 | –44.68 | 1.60 |
| E_NF135 | 39 | –44.29 | 1.57 |
| E_NR135 | 39 | –43.59 | 1.52 |
| E_NL90 | 39 | –2.02 | 0.67 |
| E_NF90 | 39 | –0.32 | 0.16 |
| E_NR90 | 39 | 1.60 | 0.52 |
| E_NL45 | 39 | 46.22 | 1.87 |
| E_NF45 | 39 | 44.97 | 1.50 |
| E_NR45 | 39 | 46.38 | 1.55 |
| E_SL135 | 39 | –45.32 | 1.49 |
| E_SF135 | 39 | –43.30 | 1.30 |
| E_SR135 | 39 | –43.72 | 1.62 |
| E_SL90 | 39 | –3.91 | 0.69 |
| E_SF90 | 39 | 0.00 | 0.09 |
| E_SR90 | 39 | 1.19 | 0.46 |
| E_SL45 | 39 | 46.67 | 1.71 |
| E_SF45 | 39 | 46.41 | 1.76 |
| E_SR45 | 39 | 48.59 | 1.69 |

facial expression: A = anger, F = fear, H = happiness, N = neutral, S = sadness

gaze direction: L = left, F = frontal, R = right

head orientation: 135 = left, 90 = frontal, 45 = right

**Supplementary Table S31.** Experiment 2: The data for emotion recognition performance (given in percent correct).

| Stimulus | N | *M* | *SE* |
| --- | --- | --- | --- |
| E_AL135 | 45 | 72.8 | 4.0 |
| E_AF135 | 45 | 83.9 | 3.7 |
| E_AR135 | 45 | 79.4 | 3.1 |
| E_AL90 | 45 | 70.0 | 3.9 |
| E_AF90 | 45 | 87.8 | 3.1 |
| E_AR90 | 45 | 89.4 | 2.2 |
| E_AL45 | 45 | 65.6 | 4.1 |
| E_AF45 | 45 | 83.3 | 3.4 |
| E_AR45 | 45 | 80.6 | 3.9 |
| E_FL135 | 45 | 63.9 | 3.7 |
| E_FF135 | 45 | 48.3 | 4.4 |
| E_FR135 | 45 | 67.8 | 3.1 |
| E_FL90 | 45 | 60.0 | 3.4 |
| E_FF90 | 45 | 47.2 | 3.9 |
| E_FR90 | 45 | 68.9 | 3.1 |
| E_FL45 | 45 | 64.4 | 3.0 |
| E_FF45 | 45 | 48.9 | 3.6 |
| E_FR45 | 45 | 66.7 | 3.6 |
| E_HL135 | 45 | 97.2 | 1.8 |
| E_HF135 | 45 | 96.7 | 1.3 |
| E_HR135 | 45 | 98.3 | 0.9 |
| E_HL90 | 45 | 97.2 | 1.2 |
| E_HF90 | 45 | 97.2 | 1.2 |
| E_HR90 | 45 | 90.6 | 2.1 |
| E_HL45 | 45 | 97.2 | 1.2 |
| E_HF45 | 45 | 97.2 | 1.2 |
| E_HR45 | 45 | 95.0 | 1.7 |
| E_NL135 | 45 | 86.1 | 2.9 |
| E_NF135 | 45 | 92.8 | 2.3 |
| E_NR135 | 45 | 90.0 | 2.0 |
| E_NL90 | 45 | 78.3 | 3.9 |
| E_NF90 | 45 | 92.2 | 2.6 |
| E_NR90 | 45 | 77.2 | 3.6 |
| E_NL45 | 45 | 77.8 | 3.7 |
| E_NF45 | 45 | 93.9 | 1.8 |
| E_NR45 | 45 | 91.1 | 2.5 |
| E_SL135 | 45 | 47.8 | 4.6 |
| E_SF135 | 45 | 64.4 | 4.1 |
| E_SR135 | 45 | 60.0 | 4.5 |
| E_SL90 | 45 | 42.8 | 4.8 |
| E_SF90 | 45 | 61.7 | 3.7 |
| E_SR90 | 45 | 60.0 | 4.7 |
| E_SL45 | 45 | 48.9 | 4.5 |
| E_SF45 | 45 | 63.9 | 3.7 |
| E_SR45 | 45 | 72.8 | 4.0 |

facial expression: A = anger, F = fear, H = happiness, N = neutral, S = sadness

gaze direction: L = left, F = frontal, R = right

head orientation: 135 = left, 90 = frontal, 45 = right

**Supplementary Table S32.** Experiment 2: The data for perceived gaze direction (given in degrees).

| Stimulus | N | *M* | *SE* |
| --- | --- | --- | --- |
| E_AL135 | 45 | –50.22 | 1.64 |
| E_AF135 | 45 | –40.86 | 1.21 |
| E_AR135 | 45 | –34.47 | 1.35 |
| E_AL90 | 45 | –28.86 | 1.48 |
| E_AF90 | 45 | –0.42 | 0.34 |
| E_AR90 | 45 | 25.36 | 1.54 |
| E_AL45 | 45 | 34.25 | 1.56 |
| E_AF45 | 45 | 45.92 | 1.61 |
| E_AR45 | 45 | 50.81 | 1.76 |
| E_FL135 | 45 | –54.83 | 1.55 |
| E_FF135 | 45 | –42.64 | 1.15 |
| E_FR135 | 45 | –31.72 | 1.56 |
| E_FL90 | 45 | –33.14 | 1.32 |
| E_FF90 | 45 | –0.47 | 0.38 |
| E_FR90 | 45 | 26.67 | 1.48 |
| E_FL45 | 45 | 24.06 | 2.03 |
| E_FF45 | 45 | 45.56 | 1.46 |
| E_FR45 | 45 | 51.58 | 1.85 |
| E_HL135 | 45 | –47.06 | 1.76 |
| E_HF135 | 45 | –40.58 | 1.24 |
| E_HR135 | 45 | –29.97 | 1.26 |
| E_HL90 | 45 | –27.97 | 1.46 |
| E_HF90 | 45 | –0.50 | 0.34 |
| E_HR90 | 45 | 24.03 | 1.61 |
| E_HL45 | 45 | 32.33 | 2.06 |
| E_HF45 | 45 | 43.14 | 1.49 |
| E_HR45 | 45 | 49.08 | 1.72 |
| E_NL135 | 45 | –48.08 | 1.75 |
| E_NF135 | 45 | –40.39 | 1.24 |
| E_NR135 | 45 | –32.53 | 1.35 |
| E_NL90 | 45 | –26.81 | 1.54 |
| E_NF90 | 45 | –0.08 | 0.17 |
| E_NR90 | 45 | 24.56 | 1.55 |
| E_NL45 | 45 | 35.56 | 1.55 |
| E_NF45 | 45 | 44.08 | 1.54 |
| E_NR45 | 45 | 49.64 | 1.93 |
| E_SL135 | 45 | –48.22 | 1.46 |
| E_SF135 | 45 | –40.36 | 1.26 |
| E_SR135 | 45 | –30.89 | 1.50 |
| E_SL90 | 45 | –26.69 | 1.45 |
| E_SF90 | 45 | –0.03 | 0.33 |
| E_SR90 | 45 | 23.94 | 1.45 |
| E_SL45 | 45 | 33.75 | 1.70 |
| E_SF45 | 45 | 43.86 | 1.51 |
| E_SR45 | 45 | 47.89 | 1.59 |

facial expression: A = anger, F = fear, H = happiness, N = neutral, S = sadness

gaze direction: L = left, F = frontal, R = right

head orientation: 135 = left, 90 = frontal, 45 = right

**Supplementary Table S33.** Experiment 2: The data for perceived head orientation (given in degrees).

| Stimulus | N | *M* | *SE* |
| --- | --- | --- | --- |
| E_AL135 | 45 | –43.33 | 1.45 |
| E_AF135 | 45 | –40.67 | 1.13 |
| E_AR135 | 45 | –41.58 | 1.53 |
| E_AL90 | 45 | –2.28 | 1.07 |
| E_AF90 | 45 | –0.03 | 0.06 |
| E_AR90 | 45 | 2.78 | 0.96 |
| E_AL45 | 45 | 46.31 | 1.72 |
| E_AF45 | 45 | 46.89 | 1.50 |
| E_AR45 | 45 | 47.47 | 1.72 |
| E_FL135 | 45 | –43.28 | 1.39 |
| E_FF135 | 45 | –43.00 | 1.08 |
| E_FR135 | 45 | –41.50 | 1.26 |
| E_FL90 | 45 | –2.14 | 1.09 |
| E_FF90 | 45 | –0.39 | 0.34 |
| E_FR90 | 45 | 3.08 | 0.83 |
| E_FL45 | 45 | 47.14 | 1.67 |
| E_FF45 | 45 | 46.75 | 1.40 |
| E_FR45 | 45 | 48.08 | 1.67 |
| E_HL135 | 45 | –41.86 | 1.37 |
| E_HF135 | 45 | –41.61 | 1.25 |
| E_HR135 | 45 | –41.28 | 1.47 |
| E_HL90 | 45 | –2.72 | 1.18 |
| E_HF90 | 45 | –0.08 | 0.23 |
| E_HR90 | 45 | 2.75 | 1.15 |
| E_HL45 | 45 | 44.08 | 1.88 |
| E_HF45 | 45 | 45.19 | 1.55 |
| E_HR45 | 45 | 47.36 | 1.59 |
| E_NL135 | 45 | –42.22 | 1.48 |
| E_NF135 | 45 | –41.56 | 1.29 |
| E_NR135 | 45 | –41.42 | 1.37 |
| E_NL90 | 45 | –1.67 | 0.90 |
| E_NF90 | 45 | –0.36 | 0.16 |
| E_NR90 | 45 | 1.81 | 0.84 |
| E_NL45 | 45 | 45.81 | 1.46 |
| E_NF45 | 45 | 45.36 | 1.64 |
| E_NR45 | 45 | 45.25 | 1.53 |
| E_SL135 | 45 | –44.53 | 1.43 |
| E_SF135 | 45 | –42.11 | 1.34 |
| E_SR135 | 45 | –39.33 | 1.39 |
| E_SL90 | 45 | –2.47 | 0.82 |
| E_SF90 | 45 | –0.11 | 0.15 |
| E_SR90 | 45 | 2.39 | 0.87 |
| E_SL45 | 45 | 44.86 | 1.58 |
| E_SF45 | 45 | 45.56 | 1.44 |
| E_SR45 | 45 | 46.42 | 1.48 |

facial expression: A = anger, F = fear, H = happiness, N = neutral, S = sadness

gaze direction: L = left, F = frontal, R = right

head orientation: 135 = left, 90 = frontal, 45 = right
